# Applications of Natural Polymers in the Grapevine Industry: Plant Protection and Value-Added Utilization of Waste

**DOI:** 10.3390/polym17010018

**Published:** 2024-12-25

**Authors:** Daniela-Ionela Toma (Sărdărescu), Doina Manaila-Maximean, Irina Fierascu, Anda Maria Baroi, Roxana Ioana Matei (Brazdis), Toma Fistos, Irina Elena Chican, Radu Claudiu Fierascu

**Affiliations:** 1 National Research and Development Institute for Biotechnology in Horticulture–INCDBH, 37 Bucuresti-Pitesti Str., 117715 Ștefănești, Romania; ionela.toma93@yahoo.com; 2 Faculty of Chemical Engineering and Biotechnology, National University of Science and Technology Politehnica Bucharest, 1-7 Gheorghe Polizu St., 011061 Bucharest, Romania; 3 Faculty of Applied Sciences, National University of Science and Technology Politehnica Bucharest, 060042 Bucharest, Romania; 4 Academy of Romanian Scientists, 3 Ilfov, 050044 Bucharest, Romania; 5 National Institute for Research & Development in Chemistry and Petrochemistry—ICECHIM Bucharest, 202 Spl. Independentei, 060021 Bucharest, Romania; irina.fierascu@icechim.ro (I.F.); anda.baroi@icechim.ro (A.M.B.); toma.fistos@icechim.ro (T.F.); irina-elena.chican@icechim.ro (I.E.C.); 6 Faculty of Horticulture, University of Agronomic Sciences and Veterinary Medicine of Bucharest, 59 Marasti Blvd., 011464 Bucharest, Romania

**Keywords:** pathogen resistance, grapevine waste valorization, biodegradable materials, bioactive compound encapsulation

## Abstract

The grapevine industry is confronted with challenges such as plant stress from environmental factors and microbial infections, alongside the need for sustainable waste management practices. Natural polymers offer promising solutions to these issues due to their biocompatibility, biodegradability, and functional versatility. This review explores the dual role of natural polymers in enhancing the grapevine industry: as protective agents against various stressors and as carriers for the delivery of valuable compounds recovered from grapevine wastes. We examine the use of natural polymers such as chitosan, alginate, and cellulose in formulating bio-based protective coatings and treatments that bolster plant resistance to abiotic stress, pathogens, and pests. Additionally, the review delves into the innovative utilization of grapevine residues, including skins, seeds, and stems, as sources of polyphenols and other bioactive compounds. These compounds can be efficiently encapsulated in natural polymer matrices for applications in agriculture, food, and pharmaceuticals. Key topics include the mechanisms of action, benefits, and limitations of natural polymer-based interventions, as well as case studies demonstrating their practical implementation in vineyards. The review also addresses future research directions, emphasizing the need for integrated approaches that enhance sustainability and economic viability in the grapevine industry.

## 1. Introduction

The grapevine industry holds a position of great economic, cultural, and agricultural importance globally, extending across a range of products including wine, table grapes, raisins, and other grape-derived goods [1]. As one of the oldest cultivated crops, grapevines have established themselves as vital to the economies of many countries, particularly in regions with climates that favor viticulture, such as Europe, the Americas, Australia, and South Africa [2]. The industry supports millions of jobs worldwide, from vineyard workers and agricultural engineers to winemakers, distributors, and retail specialists, creating extensive value chains that are deeply embedded in regional and global economies [3].

**Figure 1:**
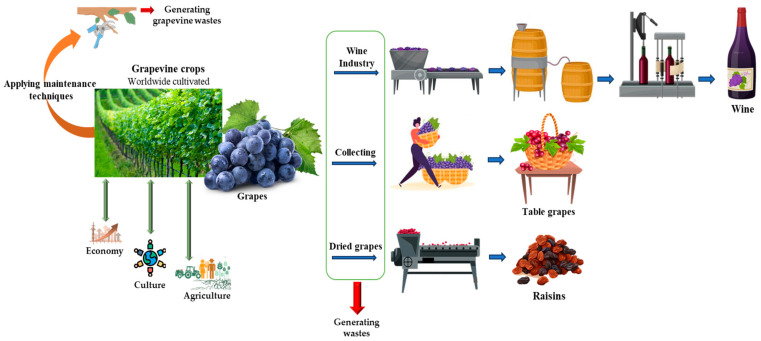
Aspects of grapevine industry.

Wine production is by far the most economically significant aspect of grapevine cultivation. The global wine industry generates substantial revenue, with demand steadily increasing across both traditional markets like Europe and newer markets in Asia and the Americas. Wine culture is historically and culturally significant in numerous countries, providing economic resilience for regions where tourism, gastronomy, and artisanal industries are tied to local vineyards [4]. Table grapes cater to a global market that prizes fresh produce as part of a healthy diet, driving demand throughout the year due to international shipping and controlled-environment agriculture [5]. Raisins and other dried grape products also hold significant market value and are increasingly sought after, contributing further to the versatility and profitability of grapevine products [6] (Figure 1).

However, the global grapevine industry faces challenges that necessitate more sustainable practices, both to safeguard the environment and to ensure long-term economic stability. Viticulture is resource-intensive, particularly regarding water and nutrient inputs, and is highly vulnerable to environmental stressors such as drought, extreme temperatures, and pest infestations. Climate change exacerbates these challenges, as warming trends and unpredictable weather events place unprecedented stress on vineyards worldwide. These conditions have direct impacts on grape quality, yield stability, and production costs. Sustainable practices in viticulture are therefore crucial not only for preserving the quality and economic value of grape-derived products but also for protecting the ecosystems within which vineyards operate [7].

Sustainability in the grapevine industry encompasses a range of practices that reduce environmental impacts and promote resource efficiency. For example, precision agriculture, which leverages technology to monitor vineyard conditions in real-time, allows for targeted interventions that conserve water, minimize fertilizer usage, and reduce chemical inputs. Biological controls and integrated pest management systems offer alternatives to conventional pesticides, helping protect beneficial species and maintain soil health. Additionally, organic and biodynamic viticulture practices are gaining traction as both consumers and producers recognize the value of environmentally conscious farming methods that prioritize biodiversity and soil vitality [8].

Waste management is another critical component of sustainability in the grapevine industry, which generates large quantities of organic waste, including grape pomace, skins, seeds, stems, and leaves. By developing processes to recover polyphenols, fibers, and other bioactive ingredients from grape waste, the industry can create new value-added products for use in agriculture, food, cosmetics, and pharmaceuticals. This approach aligns with the principles of the circular economy, wherein byproducts from one process serve as raw materials for another, thus reducing waste and maximizing resource efficiency [9].

In addition to environmental benefits, sustainable practices in the grapevine industry have significant economic advantages. By reducing reliance on costly agricultural inputs, such as water, fertilizers, and pesticides, sustainable practices lower production costs over time. Moreover, the growing consumer preference for sustainably produced goods, particularly within the premium wine market, allows producers to achieve higher price points and cultivate brand loyalty. Sustainable vineyards are also more resilient to climate and market fluctuations, making them less vulnerable to environmental and economic shocks. As such, sustainable practices in viticulture represent a convergence of ecological responsibility and economic prudence, positioning the industry for a more stable and profitable future [8].

By adopting resource-efficient, environmentally friendly methods, and pursuing value-added applications for grape-derived waste, the industry can not only mitigate its environmental footprint but also enhance its economic viability in an increasingly sustainability-conscious world. Through research, innovation, and collaborative efforts across sectors, the grapevine industry has the potential to become a model of agricultural sustainability, setting an example for other crop systems to follow [7].

The present review provides an analysis of how natural polymers can address critical challenges in the grapevine industry, particularly in plant protection and sustainable waste management. By exploring the biocompatible and biodegradable nature of natural polymers such as chitosan, alginate, and cellulose, the work highlights their effectiveness as bio-based coatings and treatments that strengthen grapevine resilience to environmental stress, pests, and pathogens. Additionally, the review discusses innovative methods to valorize grapevine waste by encapsulating bioactive compounds in natural polymer matrices, unlocking applications in agriculture, food, and pharmaceuticals. Through detailed case studies and examination of both benefits and limitations, this review emphasizes the dual potential of natural polymers to improve plant health and create value-added products from grape byproducts. Furthermore, it identifies future research directions for developing integrated, sustainable solutions to enhance both environmental and economic sustainability within the grapevine sector.

## 2. Challenges in Grapevine Industry and Potential of Natural Polymers

### 2.1. Current Challenges in Grapevine Industry

The grapevine industry, despite its longstanding agricultural and economic significance, faces a complex set of challenges that pose risks to both productivity and sustainability. As viticulture is highly dependent on environmental conditions, it is particularly susceptible to various stressors, including those arising from climate change [10]. Drought, temperature extremes, and other forms of environmental stress have become increasingly problematic for grapevine health and crop quality. These abiotic stressors can cause physiological damage, affect plant growth, and, ultimately, impair yield and grape quality, influencing the sensory attributes of wines and other grape-derived products [11,12]. Additionally, the grapevine’s sensitivity to climatic factors makes it vulnerable to shifts in weather patterns that bring unseasonal or extreme temperatures, which can disrupt flowering, berry ripening, and harvest schedules [13]. Drought, for instance, limits water availability, impairing nutrient uptake and causing cellular dehydration. This stress response leads to reduced berry size and altered sugar and acid balance, affecting both the volume and quality of the yield. Extreme heat events compound these effects by accelerating the ripening process, which can result in an imbalance in phenolic composition and flavor compounds, diminishing the quality of grapes for wine production [14].

Another significant challenge to the grapevine industry is the prevalence of microbial infections and pest infestations, which threaten vine health and productivity. Fungal diseases, such as powdery mildew, downy mildew, and *Botrytis* bunch rot, are among the most common and damaging pathogens affecting grapevines worldwide [15]. These pathogens not only reduce grape yield and quality but also necessitate frequent and intensive applications of chemical fungicides, which are costly and environmentally burdensome. Additionally, the emergence of resistant strains further complicates disease management, making it increasingly difficult to maintain healthy vineyards without significant intervention [16]. Bacterial pathogens, such as *Xylella fastidiosa*, which causes Pierce’s disease, are also a growing concern as they lead to vine death and have no effective chemical control methods [17]. Pest infestations present another layer of threat; insects such as the grapevine moth and phylloxera cause extensive damage to vines, impacting both foliage and root systems, which can lead to stunted growth, reduced productivity, and, in severe cases, vine mortality [18]. Integrated pest management strategies have been introduced to mitigate these risks, combining biological control agents, resistant rootstocks, and monitoring practices. However, managing these biological threats remains a constant challenge, as pests and pathogens continue to adapt, demanding innovative solutions for disease resistance and pest control [19].

Beyond the challenges posed by environmental stressors and biological threats, the grapevine industry also grapples with significant waste management issues. Grapevine cultivation and winemaking generate substantial volumes of organic waste, including grape pomace, skins, seeds, stems, leaves, and pruning residues. These byproducts are often generated in concentrated quantities during harvest and winemaking seasons, making their disposal an urgent but difficult task. Traditionally, grapevine waste has been disposed of or used as low-value agricultural compost or livestock feed [9]. However, as awareness of environmental impacts has grown, the industry is under pressure to adopt more sustainable waste management practices that reduce the ecological footprint of viticulture. Grape-derived waste is rich in bioactive compounds, such as polyphenols, flavonoids, dietary fiber, and seed oils, which have potential applications in food, nutraceuticals, cosmetics, and pharmaceuticals [9,20]. Thus, there is a growing interest in valorizing grapevine waste, not only to minimize waste but also to create new revenue streams. The challenge, however, lies in developing efficient and cost-effective extraction and processing methods that can recover these valuable components at an industrial scale.

**Figure 2:**
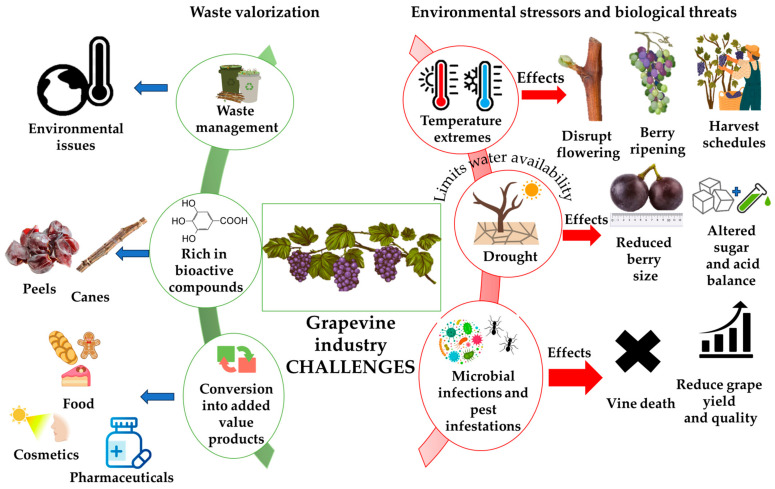
Grapevine industry challenges to be addressed by research.

The implementation of sustainable waste management solutions in viticulture is hampered by several factors, including the seasonal nature of waste generation, the high cost of extraction technologies, and the logistical challenges associated with transporting and processing large volumes of biomass [21]. Additionally, the stability and bioavailability of extracted compounds can vary, which impacts their commercial viability in different applications [9,22]. Consequently, although waste valorization offers promising avenues for sustainability, the practical and economic barriers associated with these processes need to be addressed to make them accessible to the industry at large. Figure 2 graphically presents the major challenges of the grapevine industry.

**Figure 3:**
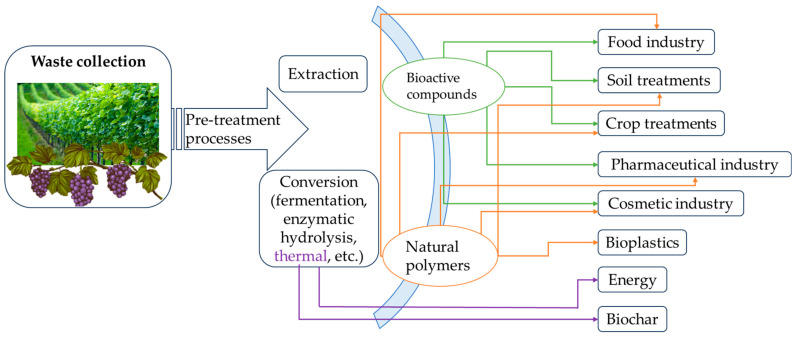
Representation of grapevine wastes valorization workflow.

The challenges faced by the grapevine industry are multi-faceted, encompassing environmental, biological, and waste management dimensions. Addressing these issues requires a holistic approach that combines advancements in biotechnology, materials science, and sustainable agricultural practices. As climate change continues to drive environmental extremes, developing drought-resistant grape varieties and optimizing water use will be essential for ensuring the resilience of vineyards. Likewise, integrated strategies that combine genetic resistance, biological controls, and monitoring systems can help manage the growing threats of pests and diseases. Finally, waste valorization represents a critical component of a more sustainable and circular grapevine industry, enabling producers to turn waste into value-added products while reducing their environmental impact (Figure 3). By embracing these approaches, the grapevine industry can better navigate its current challenges, safeguarding both its economic future and environmental responsibility.

### 2.2. Potential of Natural Polymers as Sustainable Solutions

Natural polymers represent a promising class of materials with unique properties that make them well-suited for sustainable applications in agriculture, including the grapevine industry [23]. Derived from renewable biological sources, such as plants, animals, and microorganisms, natural polymers include materials like chitosan, cellulose, alginate, pectin, and starch. These polymers are known for their biocompatibility, biodegradability, and functional versatility, qualities that align well with the growing demand for environmentally friendly solutions in agricultural production. As such, natural polymers offer a sustainable alternative to synthetic polymers and other non-biodegradable inputs, which often contribute to environmental pollution and can disrupt soil and plant health over time [24].

The biocompatibility of natural polymers is one of their most significant advantages, particularly for applications in plant protection and crop enhancement. Biocompatibility refers to the ability of these polymers to interact harmoniously with biological systems, such as plant tissues and soil microbiomes, without causing toxicity or adverse effects [25]. This property is critical in agriculture, where materials come into direct contact with plants, soil, and other components of the ecosystem. For example, when natural polymers are used as coatings for seeds, fruits, or plant surfaces, they do not introduce harmful residues, and their degradation products are typically non-toxic, allowing for a more seamless integration into existing agricultural systems [26]. Additionally, biocompatibility facilitates the use of natural polymers as carriers for bioactive compounds, such as fertilizers, pesticides, or growth enhancers, which can be delivered gradually to crops in a controlled and efficient manner [23]. This targeted delivery reduces the need for frequent reapplications and minimizes the risk of environmental contamination, contributing to more sustainable and precise farming practices.

Equally important is the biodegradability of natural polymers, which allows them to decompose into benign byproducts within a natural timescale under environmental conditions. Unlike conventional synthetic polymers, which can persist in the environment for decades and contribute to soil and water pollution, natural polymers break down relatively quickly, depending on factors such as polymer type, environmental conditions, and the presence of microbial activity [27]. This property significantly reduces the risk of environmental pollution and accumulation of plastic residues in agricultural soils, making natural polymers an ecologically sound choice for agricultural applications. Their biodegradability is particularly relevant in contexts where large volumes of material are applied directly to fields or plants, such as in the case of mulch films, protective coatings, and seed encapsulants. By degrading into non-toxic components that often serve as nutrients or organic matter for soil, natural polymers contribute to soil health and fertility, supporting the broader goals of sustainable agriculture.

In addition to biocompatibility and biodegradability, natural polymers offer a wide range of functional properties that make them highly versatile for agricultural use. For instance, polymers like chitosan, derived from chitin (a polysaccharide found in the exoskeletons of crustaceans and insect cuticles), exhibit natural antimicrobial and antifungal properties [28]. This makes chitosan particularly useful in formulating protective coatings or treatments for crops, as it can help mitigate pathogen-related losses without the need for synthetic fungicides. Similarly, alginate, extracted from brown seaweed, is known for its gel-forming ability, which is advantageous for applications that require controlled release of nutrients or other bioactive agents [29]. When used in encapsulation technologies, alginate gels can effectively protect sensitive compounds and allow for their gradual release into the plant or soil environment, enhancing nutrient availability over time and reducing nutrient losses due to leaching or volatilization [30]. Cellulose and its derivatives, obtained from plant cell walls, are valued for their mechanical strength, film-forming capacity, and compatibility with other materials, making them ideal for creating biodegradable films, mulches, and seed coatings [31].

The relevance of natural polymers in agriculture is particularly evident in applications that enhance plant resilience to environmental stressors, improve crop quality, and reduce the ecological footprint of farming practices. For example, natural polymer-based coatings and films can serve as protective barriers that help plants retain moisture, reduce water loss, and withstand fluctuations in temperature. These coatings can be applied to grapevine fruits, leaves, or other plant parts, providing a protective layer that mitigates abiotic stress and prolongs post-harvest quality [23]. By forming a breathable barrier on plant surfaces, natural polymers can also enhance the microenvironment around the plant, creating conditions less favorable for pests and pathogens while promoting plant health [32].

Natural polymers are equally promising in the context of delivering active compounds derived from agricultural byproducts or waste streams. Through encapsulation and controlled-release technologies, it can be facilitated the efficient delivery of bioactive compounds extracted from agricultural residues, such as grape pomace, seeds, and skins [33,34]. This is particularly relevant for grapevine cultivation, where byproducts are rich in polyphenols, antioxidants, and other valuable compounds that can serve as plant growth stimulants, biopesticides, or soil conditioners. Encapsulating these bioactives in natural polymers enables their gradual release, providing sustained efficacy over time and minimizing the frequency and quantity of applications [35,36]. In this way, natural polymers support the concept of circular agriculture by transforming waste into value-added inputs that contribute to plant health and productivity [9].

As the demand for sustainable agricultural practices intensifies, natural polymers present an innovative and practical solution that aligns with ecological and economic goals. They provide an alternative to conventional agricultural inputs that are often resource-intensive and environmentally detrimental, offering a pathway toward farming systems that are more in harmony with natural processes. By leveraging the unique properties of natural polymers, the agricultural sector, and specifically the grapevine industry, can advance towards more sustainable production models that prioritize environmental health, resource efficiency, and long-term agricultural resilience. As research and development in biotechnology continue to expand, the potential applications of natural polymers are likely to broaden further, contributing to an era of sustainable innovation that addresses both the challenges and opportunities within modern agriculture.

## 3. Natural Polymers Used in the Grapevine Industry

### 3.1. Overview of Common Natural Polymers and Their Properties

Natural polymers such as chitosan, alginate, and cellulose are increasingly valued in agriculture and industry for their unique properties, which align with the growing emphasis on sustainability, biocompatibility, and environmental stewardship.

The properties and applications of these natural polymers were extensively studied in the last decades, being the subject of numerous works [37,38,39]. An overview of the main natural polymers to be discussed in the current work and their properties as emerging from literature data is presented in Table 1, and further discussed in the following paragraphs.

Derived from renewable biological sources, these polymers offer a range of functionalities—from antimicrobial activity to encapsulation efficiency—that make them suitable for applications in the grapevine industry and beyond [40]. Each of these polymers brings distinct physical, chemical, and biological attributes, enabling diverse applications in plant protection, soil health, and waste valorization.

Chitosan, a polymer obtained from chitin, stands out for its antimicrobial and antifungal properties, which make it a valuable resource in agriculture as a natural protective agent. Chitin, the precursor of chitosan, is abundant in the exoskeletons of crustaceans, insects, and the cell walls of certain fungi [41]. When chemically modified through deacetylation, chitosan exhibits potent antimicrobial activity against a wide range of plant pathogens, including bacteria and fungi that commonly threaten grapevines [42]. This antimicrobial property arises from mow molecular weight chitosan’s ability to disrupt microbial cell membranes, thereby preventing the growth and spread of pathogens on plant surfaces [43]. On the other hand, chitosan was demonstrated to also exhibit extracellular effects (especially for its high molecular weight form), or both type of effects, based on the targeting site [43]. Moreover, chitosan is biodegradable, breaking down into non-toxic, naturally occurring compounds that integrate seamlessly into the soil, thus minimizing the risk of environmental contamination and promoting soil health [28]. This polymer’s antifungal action is particularly beneficial in protecting grapevines against common fungal diseases, such as powdery and downy mildew, which can significantly impact yield and grape quality. Additionally, chitosan’s film-forming properties allow it to be applied as a coating on grapevine leaves, fruits, or stems, forming a protective barrier that shields plants from environmental stressors while allowing gas exchange [44]. This protective coating not only improves plant resistance to abiotic stress, such as drought, but also enhances the shelf life of harvested grapes by reducing microbial spoilage, making chitosan an invaluable material for both pre- and post-harvest applications.

Alginate, a natural polymer extracted primarily from brown seaweed, is known for its excellent gelling, film-forming, and encapsulation capabilities, which make it highly versatile in agricultural applications [45]. Composed of mannuronic and guluronic acid units, alginate exhibits unique rheological properties, allowing it to form gels in the presence of divalent cations like calcium [46]. This gelling behavior is particularly advantageous in controlled-release formulations, where active compounds such as fertilizers, pesticides, or bio-stimulants can be encapsulated within alginate matrices and released gradually into the soil or plant system. Encapsulation with alginate not only protects these compounds from degradation but also facilitates their sustained release, enhancing their efficacy while reducing the frequency of application [47]. The film-forming ability of alginate is also notable, enabling the creation of biodegradable and breathable films that can be applied as mulch or protective coatings on plant surfaces [48]. These films help retain soil moisture, prevent weed growth, and protect plants from pests, offering a more sustainable alternative to traditional plastic mulches. Additionally, alginate’s biocompatibility ensures that it does not interfere with plant physiology, making it suitable for use in sensitive agricultural applications. In the grapevine industry, alginate-based coatings and gels can be used to encapsulate bioactive compounds extracted from grape waste, allowing for the efficient utilization of these byproducts in soil enrichment or plant growth enhancement [49]. Through its gelling, film-forming, and encapsulation properties, alginate demonstrates exceptional potential as a multifunctional material in sustainable agriculture.

Cellulose, the most abundant organic polymer on Earth, is derived from plant cell walls and is highly valued for its mechanical strength, biocompatibility, and biodegradability. Its robust molecular structure, composed of β-D-glucose units linked by β-1,4-glycosidic bonds, gives cellulose high tensile strength, making it an ideal candidate for applications that require durable and resilient materials [50]. Cellulose and its derivatives, such as carboxymethyl cellulose (CMC) and hydroxypropyl cellulose (HPC), are commonly used in the formulation of biodegradable films and coatings that can be applied to plant surfaces for protection against environmental factors [51]. The biocompatibility of cellulose ensures that it can be safely used in direct contact with plants and soil without causing toxicity or adverse effects on plant growth [52]. This attribute is particularly relevant in agriculture, where materials must interact with plant tissues and soil ecosystems in a non-disruptive manner. Cellulose-based materials degrade over time, breaking down into organic components that contribute to soil health by adding organic matter, thus supporting the principles of sustainable agriculture [53]. In the context of grapevine cultivation, cellulose derivatives can be used to create mulch films or protective covers that shield plants from extreme temperatures, retain moisture, and suppress weed growth [54,55]. Furthermore, cellulose’s high surface area and porosity make it an excellent carrier for bioactive compounds, enabling the delivery of nutrients, pesticides, or plant growth regulators in a controlled manner [56]. This controlled release capability allows for the gradual availability of active compounds, optimizing their impact on plant health while reducing the need for repeated applications.

When compared with synthetic polymers, the natural polymers generally have a lower environmental footprint due to their renewable sourcing and biodegradability. However, their environmental benefits depend on sustainable farming and processing practices [57]. In the following paragraphs we will briefly discuss the advantages of the natural polymers in terms of environmental footprint, compared with the synthetic polymers.

Source: the natural polymers are derived from renewable biomass like plants, animals, or microorganisms, leading to low carbon emissions from raw material sourcing. As adverse effects, their sourcing could involve land-use changes, deforestation, or water-intensive agriculture [58]. On the other hand, synthetic polymers are derived from petrochemicals, a non-renewable resource, leading to high greenhouse gas emissions during extraction and refining; the dependence on fossil fuels contributes to resource depletion [59].

Manufacturing process: the natural polymers obtaining is often less energy-intensive, but may require extensive processing (e.g., spinning, chemical treatments), which could involve significant water use and potential release of organic pollutants [60]. Manufacturing of synthetic polymers is typically energy-intensive, involving polymerization and refining. Emissions include greenhouse gases, volatile organic compounds (VOCs), and hazardous waste [61].

Life-cycle durability: natural polymers are biodegradable under natural conditions. They have limited durability compared to synthetic counterparts, and a lower accumulation in landfills or oceans, but may release CO₂ during degradation [27]. Synthetic polymers are generally non-biodegradable, persisting for centuries in the environment. They are however highly durable, a characteristic often desirable for long-term applications. Synthetic polymers are a major contributor to plastic pollution and microplastic formation [27].

Carbon footprint: for natural polymers, it depends on agricultural practices, energy used in processing, and transportation. Most processes are often carbon-neutral or near-neutral if sustainable practices are followed. Their synthetic counterparts have a high carbon footprint due to energy-intensive production and fossil fuel dependency, while end-of-life scenarios (incineration or landfilling) contribute to additional emissions [27].

Water footprint: agriculture-based natural polymers have significant water footprints (e.g., irrigation and dyeing), also presenting a pollution risks from pesticide and fertilizer use. Synthetic polymers have a lower water use in production but can cause significant water pollution from industrial discharges [62].

Waste and recycling: natural polymers tend to degrade naturally but recycling is less common due to chemical variability. Composting is a viable end-of-life option for some natural polymers. Synthetic polymers have a low recycling rate (~9% globally for plastics). Mechanical or chemical recycling is energy-intensive and often economically unfeasible [27].Collectively, these natural polymers—chitosan, alginate, and cellulose—offer a range of properties that support sustainable practices in the grapevine industry. Their inherent biodegradability ensures that they do not contribute to soil or water pollution, while their biocompatibility allows them to interact harmoniously with plant and soil systems. Each polymer’s unique properties—chitosan’s antimicrobial action, alginate’s encapsulation and film-forming abilities, and cellulose’s mechanical strength and compatibility—provide multiple pathways for enhancing plant protection and improving the efficiency of agricultural inputs. As natural, renewable, and multifunctional materials, these polymers align with the goals of sustainable viticulture, offering innovative solutions that can enhance crop resilience, reduce environmental impact, and promote the circular utilization of agricultural byproducts.

### 3.2. Functional Properties of Natural Polymers Relevant to Grapevine Applications

Natural polymers (e.g., chitosan, alginate, or cellulose) offer a combination of functional properties that make them highly suitable for applications in the grapevine industry, particularly in plant protection, enhancement of plant resilience, and sustainable waste utilization. Among these properties, biocompatibility with plants, film-forming capability, and their ability to carry and deliver active agents are central to their utility. These characteristics allow natural polymers to interact seamlessly with grapevine tissues, enhance plant health, and introduce bioactive compounds in a controlled and efficient manner, supporting sustainable viticulture practices that prioritize environmental responsibility [63].

The biocompatibility of natural polymers is a fundamental property that underpins their broad use in agriculture, especially in direct applications on plants. Biocompatibility refers to a polymer’s capacity to interact with biological systems—such as plant cells and soil microbiota—without causing adverse effects. This compatibility is critical in grapevine cultivation, where materials applied to leaves, stems, or fruits must not interfere with physiological processes or plant health. Unlike synthetic polymers, which may leave harmful residues or persist in the environment, natural polymers degrade into non-toxic byproducts that are easily assimilated into the soil, thus contributing to nutrient cycling and soil fertility [64]. For instance, chitosan demonstrates natural antimicrobial and growth-stimulating properties while remaining safe for grapevine tissues and soil ecosystems [65]. When applied as a coating or soil additive, chitosan not only provides protection against pathogens but also promotes beneficial interactions in the rhizosphere, enhancing root health and nutrient absorption [66]. Alginate and cellulose derivatives likewise demonstrate excellent biocompatibility, making them ideal candidates for encapsulating nutrients, pesticides, or plant growth regulators without disrupting plant function or compromising soil health [67].

The film-forming ability of these natural polymers is another key property that enhances their utility in grapevine applications. Film-forming capacity allows polymers to create protective layers over plant surfaces, which serve multiple functions: reducing water loss, shielding plants from UV radiation and temperature fluctuations, and forming a barrier against pathogens and pests. For example, chitosan and alginate can be formulated into thin, breathable films that adhere to leaves or fruits, creating a microenvironment that helps maintain optimal humidity and temperature while restricting entry to harmful microorganisms [68]. Such films are especially beneficial for grapevines, which are sensitive to fluctuations in environmental conditions [69]. Alginate, with its unique gel-forming properties in the presence of calcium ions, can produce stable films that adhere well to plant surfaces, acting as a protective layer that is biodegradable and does not hinder gas exchange [70]. Cellulose is also utilized in creating durable films or mulches that provide lasting protection, particularly in vineyard rows, where soil temperature regulation and moisture retention are essential for vine health and productivity [71,72]. These films can also be employed post-harvest, as protective coatings that extend the shelf life of grapes by reducing respiration rates and microbial spoilage, thereby preserving fruit quality during storage and transport [73].

The ability of natural polymers to serve as carriers for active agents is another property that adds substantial value to their application in grapevine management. This carrier capacity allows for the controlled delivery of bioactive compounds, such as nutrients, growth hormones, pest deterrents, or antioxidants derived from grapevine waste, directly to target sites within the plant or soil. Encapsulation techniques, particularly using alginate and cellulose, enable the loading of these active agents into polymer matrices, creating controlled-release systems that gradually deliver their contents over time [47]. This controlled release reduces the frequency of applications and minimizes wastage, allowing for sustained protection or enhancement without overloading the plant or soil with chemicals. For example, alginate gels, due to their excellent encapsulation capabilities, are used to entrap bioactive compounds extracted from grape waste, such as polyphenols, which can then be applied to soils to enhance microbial activity or be released into grapevine tissues to improve resilience against environmental stress [49]. Chitosan, with its inherent antimicrobial properties, can act as a carrier for pesticides or fungicides, further amplifying its protective effects against pathogens like powdery and downy mildew, common diseases in vineyards [74]. By binding these agents within a natural polymer matrix, these compounds can be precisely targeted to areas of need, reducing the reliance on synthetic agrochemicals and thereby aligning with sustainable viticulture goals.

In addition to these individual functionalities, the synergistic effects of these polymers further enhance their suitability for grapevine applications. When combined, the biocompatibility, film-forming capacity, and active-agent carrier properties of natural polymers provide an integrated approach to plant care, which addresses both plant protection and environmental sustainability [68]. By offering protection against abiotic stresses, reducing microbial threats, and facilitating nutrient and bioactive delivery, these polymers support healthy grapevine growth and productivity across various stages of cultivation and post-harvest handling. Moreover, their natural degradation in the soil adds organic matter, supporting soil structure and fertility, which benefits grapevines over time by promoting a balanced and resilient ecosystem. As the grapevine industry increasingly seeks methods to reduce environmental impact, these functional properties of natural polymers present a comprehensive solution, advancing sustainable practices that are both effective and ecologically responsible.

## 4. Applications in Plant Protection

### 4.1. Natural Polymer-Based Coatings and Treatments for Grapevine Protection

In the context of plant protection, natural polymer-based coatings and treatments present a promising solution for enhancing grapevine resilience against a range of environmental stressors. These bio-based coatings, derived from natural polymers like chitosan, alginate, and cellulose, have the potential to serve as an effective barrier that safeguards grapevines from abiotic and biotic stresses. Their application in viticulture is driven by their biocompatibility, biodegradability, and protective properties, which make them ideal for reducing water loss, buffering against temperature variations, blocking harmful UV radiation, and inhibiting pathogen entry. These coatings can be applied to the leaves, fruits, and stems of grapevines, providing both immediate and prolonged benefits that contribute to overall plant health and productivity.

Bio-based coatings and film formulations specifically designed for grapevine protection involve the use of naturally derived polymers that can be applied as thin films or coatings over plant surfaces [75]. These films act as physical barriers while also enhancing the plant’s microenvironment, enabling a more controlled response to stress factors. When applied to grapevines, these coatings create a microfilm that adheres to the plant surface, forming a breathable yet protective layer that helps retain moisture and prevent desiccation under drought conditions. The film-forming ability of polymers like chitosan and alginate is particularly valuable here, as it allows for the creation of coatings that are both flexible and resilient, adhering to the uneven surfaces of leaves and fruits without compromising the plant’s natural processes, such as gas exchange and photosynthesis. Moreover, these bio-based films can be engineered to include additional functional compounds, such as antioxidants, antimicrobial agents, or UV protectants, which further enhance the protective effect and contribute to grapevine health.

One of the primary mechanisms by which these natural polymer-based coatings protect grapevines from abiotic stress is through water retention [76]. Given that grapevines are often exposed to variable and sometimes extreme environmental conditions, maintaining adequate hydration within plant tissues is crucial. When applied to grapevine leaves and fruits, natural polymer coatings reduce transpiration by creating a semi-permeable barrier that slows down water loss, effectively allowing the plant to conserve moisture under drought conditions. This water-retention capacity is particularly useful during dry seasons or in regions where water resources are limited. Chitosan-based coatings, for instance, have been shown to reduce the rate of water loss by forming a protective layer that minimizes direct exposure of plant tissues to the atmosphere, helping grapevines to withstand prolonged dry periods with minimal physiological stress [65]. Additionally, alginate and cellulose derivatives can be used to create films that enhance the plant’s ability to retain moisture, reinforcing its natural defenses against dehydration [71].

Temperature fluctuations also pose a significant challenge in grapevine cultivation, particularly during sudden heat waves or cold snaps that can disrupt growth and compromise fruit quality. Natural polymer coatings act as thermal buffers, moderating the temperature of the plant surface by insulating it from rapid temperature shifts. This effect is achieved as the film’s composition helps to stabilize the plant’s microenvironment, reducing the rate at which temperature changes impact the plant tissue. For instance, in warmer conditions, these coatings can reflect a portion of solar radiation, thereby reducing heat absorption and helping maintain optimal leaf and fruit temperatures. Conversely, in cooler environments, the coating may serve as an insulating layer, minimizing the impact of cold air on plant tissues and preventing damage to sensitive buds or leaves. In this way, natural polymer-based films provide grapevines with a layer of protection that shields against temperature extremes, enhancing resilience across seasons and supporting more consistent growth [26].

Protection from ultraviolet (UV) radiation is another critical function of natural polymer coatings, as excess UV exposure can cause cellular damage, impair photosynthesis, and degrade fruit quality. Natural polymers such as chitosan can be combined with UV-blocking agents to form UV-protection coatings [77]. This is particularly important in high-altitude or sunny regions where grapevines are exposed to intense sunlight, as prolonged exposure to UV can lead to oxidative stress and impact the overall health of the plant. By absorbing or reflecting harmful UV rays, these coatings can prevent cellular damage, reduce oxidative stress, and support optimal photosynthetic activity, ultimately contributing to improved yield and fruit quality.

Although the exact molecular mechanism between natural polymers and grapevine interaction is not fully elucidated, a pathway for increasing the grapevine resistance to difference environmental stressors was proposed: the natural polymers could induce the synthesis of secondary metabolites which Elicitors are the molecules that induce the secondary metabolite synthesis in plants, thus eliciting pathways for improved plant response [65].

In addition to abiotic stress protection, natural polymer-based coatings act as a barrier against pathogens [78], further enhancing grapevine resilience. Chitosan, in particular, exhibits inherent antimicrobial and antifungal properties that make it effective in reducing infections caused by pathogens like powdery mildew and downy mildew, both of which are common in vineyards. When applied to grapevine surfaces, chitosan forms a film that inhibits microbial colonization, physically blocking pathogens from penetrating plant tissues. Furthermore, chitosan can stimulate plant defense mechanisms by inducing the production of defense-related enzymes, which fortify the plant’s resistance to infection [79]. This dual action—acting as both a barrier and a biochemical inducer—makes chitosan-based coatings a highly effective tool in vineyard disease management. Alginate and cellulose-based coatings can also serve as physical barriers, reducing the surface moisture that many fungal pathogens rely on for spore germination and spread. This decrease in surface moisture creates an unfavorable environment for pathogens, further contributing to disease resistance and reducing the need for chemical fungicides.

Overall, the applications of natural polymer-based coatings in the grapevine industry are multifaceted, addressing key environmental and biotic challenges that grapevines face throughout their lifecycle. By improving water retention, stabilizing temperature, providing UV protection, and inhibiting pathogen infiltration, these bio-based treatments offer a holistic approach to plant protection that not only supports grapevine health but also aligns with sustainable agriculture practices. As natural polymers are biodegradable and derived from renewable sources, their use minimizes environmental impact, positioning them as a viable alternative to conventional synthetic coatings and treatments. Through their multifaceted functionality, natural polymer-based coatings thus contribute to both the immediate protection of grapevines and the long-term sustainability of viticulture.

### 4.2. Mechanisms of Action Against Pathogens and Pests

Natural polymers such as chitosan and alginate offer distinct antimicrobial and functional properties that make them invaluable tools for managing pathogens and pests in the grapevine industry. The mechanisms by which these polymers inhibit pathogens involve both direct antimicrobial effects and the capacity to serve as carriers for bioactive compounds, enhancing their protective efficacy. Through these mechanisms, natural polymers provide a sustainable and eco-friendly alternative to synthetic chemicals, addressing growing concerns about environmental impact and residue accumulation in the soil and on crops. These polymers have demonstrated their effectiveness in a variety of case studies, underscoring their role in sustainable viticulture and plant health management.

Chitosan is one of the most extensively studied natural polymers in agriculture, largely due to its inherent antimicrobial properties [28]. The antimicrobial action of chitosan is multifaceted and includes both direct effects on microbial cell integrity and indirect stimulation of the plant’s own defense mechanisms. Chitosan’s antimicrobial activity arises from its positive charge, which interacts with the negatively charged components of microbial cell membranes, the exact mechanism being presented in other review works [80]. This interaction disrupts the structural integrity of the cell membrane, leading to increased permeability and eventual cell death. Chitosan’s efficacy against a wide range of pathogens, including bacteria, fungi, and viruses, has been well-documented [43,80,81]. Chitosan has shown particular promise in managing fungal infections, such as those caused by *Botrytis cinerea*, the pathogen responsible for gray mold [82], and *Plasmopara viticola*, which causes downy mildew in grapevine (the treatment reducing the disease severity by 30%, on average, on canopy) [83]. When applied as a coating or foliar spray, chitosan forms a protective barrier on the grapevine’s surface, inhibiting pathogen attachment and reducing the likelihood of infection. Additionally, chitosan has been shown to trigger plant defense responses by inducing the production of phytoalexins and other defense-related compounds, which further enhance the plant’s ability to resist infection [84].

Alginate, another naturally occurring polymer primarily extracted from brown algae, exhibits significant antimicrobial and film-forming properties that make it suitable for pathogen control in vineyards. Although alginate does not possess inherent antimicrobial properties as pronounced as chitosan, it acts as an effective matrix for incorporating and delivering antimicrobial agents, such as essential oils and fungicides [85]. By encapsulating these active agents within an alginate matrix, the compounds are protected from environmental degradation and are gradually released, maintaining an effective concentration over an extended period. This slow-release mechanism minimizes the need for frequent applications, reducing labor and material costs while ensuring continuous protection for the grapevines. Alginate-based formulations have been successfully used in other crop systems for pathogen management [86,87], suggesting a good potential for grapevine applications as well. Studies have shown that essential oils such as thymol and eugenol, known for their antimicrobial properties, are highly effective against common fungal pathogens when encapsulated in alginate [88]. These natural compounds can target and disrupt the cellular functions of fungi and bacteria while remaining safe for grapevine tissues, thus providing a targeted, low-toxicity approach to pest and pathogen management.

Several case studies illustrate the practical applications of natural polymers in reducing pathogen loads in grapevines. For instance, a study conducted on vineyards in southern Europe demonstrated that chitosan treatment can reduce the incidence of downy mildew. In the study, grapevine leaves pre-treated with chitosan were infected by *Plasmopara viticola* sporangia, the treatment supporting a significant reduction in the incidence of fungal attack, compared with the untreated leaf discs, in all the experimental variants (average incidence of the attack: 21.6% for the chitosan treatment, compared with 100% for control, 48 h after the first treatment, 36.6% for the treated samples, compared with 98.3% for control, 72 h after the second treatment, 55.5% for treated samples, compared with 78.3% for control, 72 h after the third treatment, respectively). The recorded level of efficacy allowed the authors to propose chitosan as a biological adjuvant for downy mildew defense [89]. Other authors, evaluated irradiated chitosan with *Bacillus subtilis* and *Trichoderma viride* and pesticides for disease management (against downy mildew on grapevine cv. Thompson seedless). According to the presented conclusions, irradiated chitosan and *Bacillus subtilis* presented a synergistic effect, efficiently reducing the fungal attack (percent disease index 19.83%, compared with 53.84% for the untreated crop), thus proposing chitosan as a multiple stress tolerance enhancing agent [90].

Another example comes from alginate applications combined with essential oils to manage *Colletotrichum acutatum* infestation (causing ripe rot in grapes) [91]. The study evaluated the efficacy of a coating based on calcium-alginate (0.5% w/v) and cassia oil (1.25 μL/mL) to prevent the rotting of the grapes. According to the presented results, the application of the proposed combination led to the protection of the fruits (no rotten fruits being observed after 20 days of storage, compared with the untreated samples, which were completely rotten after ten days of storage) [91].

The role of natural polymers in delivering active compounds—such as essential oils, biofungicides, or nutrients—represents a critical aspect of their utility in grapevine protection. These polymers act as carriers that encapsulate bioactive molecules, safeguarding them from premature degradation and ensuring their release at the desired site and rate. This encapsulation and controlled release not only improve the efficacy of the active agents but also extend their period of action, reducing the frequency of applications needed for effective pathogen control. Chitosan, for instance, can be used to deliver copper-based fungicides in low concentrations, which are traditionally applied in higher amounts. Encapsulating copper in chitosan not only reduces the metal’s potential phytotoxicity but also enhances its efficacy by targeting pathogen cells more directly [92]. Additionally, chitosan can facilitate the delivery of other bioactive compounds, such as plant growth regulators or micronutrients [93], to promote overall plant health and resilience against biotic stressors.

In a similar manner, alginate gels have been explored as carriers for volatile essential oils, which are naturally antimicrobial but prone to volatilization and degradation [94]. By encapsulating these oils in alginate, their release is controlled, allowing for a steady diffusion of active compounds over time. Studies have demonstrated that encapsulated essential oils can be an efficient antifungal agent against grapevine pathogens [95]. This controlled-release mechanism ensures that the oils remain active longer and exert their effects more consistently, thus maximizing the benefits of natural antimicrobial agents while minimizing the risk of phytotoxicity. Furthermore, cellulose and its derivatives can serve as a matrix for various bioactive compounds, creating coatings that combine physical protection with targeted delivery of antimicrobial or growth-promoting agents [96]. These cellulose-based films can be loaded with active compounds that act against pathogens, providing a dual function of physical and biochemical protection for grapevines [97].

Overall, the antimicrobial properties of natural polymers like chitosan, their role as carriers for bioactive compounds, and their efficacy highlight their potential for sustainable pathogen and pest management in the grapevine industry. As alternatives to synthetic chemicals, these polymers provide targeted and environmentally friendly solutions that are aligned with the principles of sustainable agriculture. Through the combined effects of direct antimicrobial activity, induction of plant defenses, and controlled release of active agents, natural polymer-based treatments offer a comprehensive approach to protecting grapevines from pathogens and pests while reducing chemical inputs and supporting soil and environmental health.

### 4.3. Advantages and Limitations of Using Natural Polymers in Plant Protection

The use of natural polymers in plant protection offers notable advantages, particularly for sustainable agriculture, but also presents specific challenges that must be addressed to fully realize their potential. These polymers, including chitosan, alginate, and cellulose derivatives, are increasingly recognized for their biodegradability, non-toxic nature, and minimal environmental impact, which stand in contrast to many synthetic agrochemicals [98]. Their integration into plant protection systems aligns with the growing demand for sustainable and eco-friendly agricultural practices, making them highly attractive for viticulture, where consumer and regulatory pressures are intensifying the push for greener solutions. However, the practical implementation of natural polymer-based treatments also encounters challenges, including cost, optimization for effective application, and the need for scalable solutions that are feasible for commercial agricultural systems.

One of the primary benefits of natural polymers is their biodegradability, which ensures that they decompose naturally in the environment, leaving no harmful residues. This characteristic is particularly significant for the grapevine industry, where repeated applications of synthetic chemicals have raised concerns about long-term soil and water contamination. Unlike synthetic polymers or conventional pesticides, natural polymers are derived from renewable biological sources, such as crustacean shells for chitosan, algae for alginate, and plant materials for cellulose. As a result, these materials decompose into non-toxic byproducts that do not accumulate in soil or groundwater, preserving the integrity of ecosystems surrounding vineyards. This biodegradability is also advantageous in terms of regulatory compliance, as natural polymers meet or exceed the standards set by many environmental and agricultural agencies, reducing the barriers to their adoption in organic and eco-certified production systems.

The non-toxic nature of natural polymers is another crucial benefit, both for plant health and for the safety of workers and consumers. In the context of grapevine cultivation, where residues from chemical treatments can persist on fruits, leaves, and stems, the use of natural polymers offers a safer alternative that poses minimal risks to human health. Chitosan, for example, has been extensively studied for its safety profile and is approved for agricultural and food applications [68]. This non-toxic profile also extends to the broader environment, as natural polymers do not leach harmful compounds into the soil or aquatic systems, reducing the impact on non-target organisms such as beneficial insects, soil microbiota, and aquatic life [23]. Moreover, as consumer demand for residue-free and organic products grows, natural polymers provide a pathway for grape producers to meet these expectations, supporting product differentiation and market access.

The environmental impact of natural polymers is also markedly lower than that of many conventional agrochemicals. Synthetic pesticides, fungicides, and herbicides often have complex environmental consequences, contributing to issues such as pollution, biodiversity loss, and disruption of soil health [99]. By using natural polymers, the grapevine industry can move towards practices that have minimal ecological footprints. For example, treatments based on natural polymers do not disrupt microbial communities in the soil, which play an essential role in nutrient cycling and overall soil fertility [100]. Additionally, by reducing dependency on synthetic chemicals, natural polymer-based treatments contribute to the preservation of biodiversity within vineyard ecosystems, supporting a more resilient and self-sustaining agricultural environment.

Despite these substantial benefits, there are challenges associated with the use of natural polymers in plant protection that must be addressed to ensure their viability on a large scale. One of the primary limitations is cost, as the extraction and processing of natural polymers can be relatively expensive compared to mass-produced synthetic chemicals. For example, the extraction of chitosan from crustacean shells involves a series of chemical treatments and purifications that can drive up production costs [101]. Similarly, alginate and cellulose require specific processing steps to yield materials with the necessary properties for agricultural applications [102,103]. While the benefits of natural polymers are considerable, the higher cost may limit their accessibility, particularly for small-scale vineyards or those operating with narrow profit margins. To overcome this, ongoing research is exploring ways to reduce production costs, such as optimizing extraction techniques and developing alternative sources, such as microbial production of biopolymers.

Another challenge lies in the need for optimization to enhance the effectiveness of natural polymers in real-world vineyard conditions. Unlike synthetic agrochemicals that are specifically engineered for targeted action, natural polymers may vary in their efficacy depending on factors such as environmental conditions, polymer composition, and application methods. For instance, while chitosan has demonstrated strong antifungal properties in laboratory settings, its effectiveness in field applications may be influenced by variables like humidity, temperature, and UV exposure. To address this, researchers are working to refine formulations and application techniques, such as incorporating additives or modifying polymer structures to improve stability and efficacy under variable conditions. Encapsulation of active compounds within polymer matrices is another strategy to enhance effectiveness, allowing for controlled release and prolonged action, which is particularly useful in fluctuating vineyard climates.

Scalability is an additional consideration that must be addressed to integrate natural polymers into large-scale viticulture effectively. While small, controlled environments such as greenhouses or research vineyards have successfully employed natural polymer treatments, the transition to large vineyard operations presents logistical challenges. Scaling up requires the development of application methods that are both efficient and compatible with existing agricultural practices. For instance, polymer-based coatings may need to be reformulated to ensure they can be applied using standard spraying equipment or other mechanized tools used in vineyards. Additionally, the timing and frequency of applications must be optimized for the needs of large-scale grapevine cultivation, balancing efficacy with practical labor and cost considerations. The development of concentrated formulations or premixed solutions that simplify the application process is one potential solution to improve scalability, making natural polymer-based treatments more feasible for commercial vineyards.

Overall, while natural polymers present some challenges, the advantages they offer make them a compelling choice for sustainable plant protection in the grapevine industry. Their biodegradability, non-toxic nature, and low environmental impact align with the principles of sustainable agriculture, and they provide an alternative to synthetic chemicals that is increasingly favored by consumers and regulators alike. Addressing cost, optimization, and scalability issues will be essential for their broader adoption, and ongoing research is expected to bring advancements that make natural polymers more accessible and effective. By overcoming these challenges, the grapevine industry can leverage the unique properties of natural polymers to protect crops in a way that supports long-term agricultural sustainability, soil health, and biodiversity, ultimately benefiting both producers and the environment.

## 5. Value-Added Utilization of Grapevine Waste Using Natural Polymers

### 5.1. Types and Composition of Grapevine Waste

The grapevine industry generates substantial quantities of byproducts, primarily in the form of grape skins, seeds, and stems, each of which presents valuable opportunities for value-added utilization. These residues, often treated as waste, are rich in bioactive compounds with applications across agriculture, food, pharmaceuticals, and cosmetics, presenting a compelling case for their recovery and reuse [9]. The utilization of these grapevine byproducts not only addresses issues of waste management but also supports a more sustainable, circular economy within the industry. By harnessing natural polymers, the functional compounds in grapevine residues can be effectively encapsulated, preserved, and delivered for various uses, enhancing both the stability and bioavailability of these compounds while contributing to environmentally responsible production practices [104].

Grapevine waste is produced primarily during the winemaking process, where large volumes of grape skins, seeds, and stems are separated from the juice and often discarded. Each of these byproducts has a distinct composition and a unique profile of bioactive compounds [105]. Grape skins, for example, constitute a significant portion of the pomace left after pressing, and are notable for their high concentrations of polyphenols, particularly anthocyanins, which contribute to the pigmentation of red grapes [106]. These compounds are renowned for their antioxidant properties, which have been linked to various health benefits, including cardiovascular protection and anti-inflammatory effects [107]. Additionally, grape skins contain other beneficial polyphenols such as flavonoids and tannins, which contribute not only to health-promoting properties but also have preservative effects in food and cosmetic applications due to their ability to combat oxidative processes [108].

Grape seeds, another major component of grape pomace, are similarly rich in polyphenolic compounds. Proanthocyanidins, a type of condensed tannin found abundantly in grape seeds, have been shown to possess strong antioxidant properties and are associated with a wide range of bioactive effects, including anti-inflammatory and anti-carcinogenic activities [109]. In addition to polyphenols, grape seeds contain essential fatty acids such as linoleic acid, which is beneficial for skin health and is therefore of interest in the cosmetics industry. The nutritional profile of grape seeds also includes proteins and dietary fibers, making them suitable for various food applications as well [110]. Given the stability of these compounds when properly encapsulated or preserved, grape seeds are a prime candidate for value-added products in both health and wellness industries, where their bioactivity can be maintained and leveraged for functional benefits [111].

Grapevine stems, though less commonly utilized, are also rich in bioactive compounds. While stems contain lower concentrations of polyphenols than skins and seeds, they provide other valuable constituents, including lignin, cellulose, and various organic acids [112,113]. Stems also contain resveratrol, a compound that has received considerable attention for its potential anti-aging and anti-cancer properties [114]. Although present in smaller amounts than in skins, the presence of resveratrol in stems adds value to these byproducts, making them suitable for extraction and concentration. In addition to their bioactive content, stems provide a rich source of structural fibers, which have potential applications in biodegradable materials, reinforcing the use of natural polymers in the processing and enhancement of these residues [115].

The nutritional and chemical composition of grapevine residues is distinguished not only by its richness in polyphenols but also by a variety of other bioactives, including fibers, organic acids, and proteins, which can be stabilized and concentrated using natural polymer matrices [105]. Polyphenols, for instance, are sensitive to environmental factors like heat, light, and oxygen, which can degrade their bioactivity [116]. Natural polymers can be employed to encapsulate these sensitive compounds, forming protective layers that prevent oxidation and degradation. By using these polymers to create microcapsules or films, the stability of polyphenolic compounds is enhanced, allowing for a controlled release of antioxidants in agricultural or cosmetic applications [117]. This approach not only preserves the bioactivity of the compounds but also allows for precise application, enabling the grapevine industry to tap into new markets for functional bioactive products derived from what would otherwise be discarded waste.

The dietary fibers present in grapevine byproducts, particularly in skins and seeds, offer additional value due to their functional properties in food and health products. Fibers extracted from grape skins can be used as natural thickeners, stabilizers, or even as prebiotic agents in dietary formulations, enhancing gut health by promoting beneficial microbial growth [118,119]. The high content of insoluble fiber in these residues supports digestive health, while the polyphenolic compounds contribute to their antioxidant properties, allowing for the development of functional food products with dual health benefits. The combination of dietary fiber and bioactive compounds positions grapevine waste as a valuable resource for the health food industry, where natural polymers can be used to encapsulate or coat these fibers, ensuring prolonged stability and enhancing their integration into various food products.

Natural polymers can further enhance the utility of grapevine residues by serving as effective carriers for bioactive compounds in pharmaceutical and cosmetic applications. For instance, the encapsulation of grape seed polyphenols within alginate or chitosan can create a sustained-release system for topical applications, providing antioxidant benefits over extended periods when used in skincare products [120,121]. This encapsulation not only protects the polyphenols from rapid degradation but also facilitates a controlled delivery to the skin, which is crucial for maintaining efficacy in cosmetic formulations. Similarly, the encapsulation of resveratrol in formulations based on natural polymers (such as cationic chitosan-coated or anionic alginate-coated poly(d,l-lactide-co-glycolide) nanoparticles) [122] provides a stable formulation that can be incorporated into anti-aging creams or serums, where it can contribute to skin repair and rejuvenation, or for oral delivery [123]. The use of natural polymers as delivery systems for these compounds enhances their functional properties and supports the development of high-value products with lasting effects.

In addition to encapsulation for bioactivity preservation, the structural properties of natural polymers allow them to interact synergistically with grapevine residues to form composite materials. For instance, combining cellulose fibers (which are found in grapevine stems) with chitosan can produce biodegradable films suitable for packaging or agricultural applications, where these materials offer durability, flexibility, and environmental safety [124]. Such films could be applied directly to crops as protective layers or used in packaging to extend the shelf life of perishable goods. The integration of grapevine-derived fibers with natural polymers aligns with the principles of green chemistry, reducing reliance on non-renewable materials and providing an eco-friendly alternative to conventional plastics.

The use of grapevine waste, facilitated by natural polymer technologies, exemplifies a sustainable approach that maximizes resource utilization while minimizing environmental impact. By transforming byproducts into valuable commodities, the grapevine industry can reduce waste disposal challenges and create new revenue streams, supporting both economic and environmental sustainability. The implementation of natural polymer-based systems for the encapsulation, preservation, and application of grapevine-derived bioactives represents a promising pathway toward a more circular economy in viticulture, where every part of the grape is utilized efficiently.

### 5.2. Extraction of Bioactive Compounds from Grapevine Waste

The extraction of bioactive compounds from grapevine waste presents a transformative opportunity for industries spanning agriculture, food, cosmetics, and pharmaceuticals. Grapevine byproducts, particularly grape skins, seeds, and stems, are rich sources of valuable compounds, including polyphenols, flavonoids, dietary fibers, and essential fatty acids. These compounds are known for their potent bioactive properties, such as antioxidant, anti-inflammatory, and antimicrobial effects, making them attractive ingredients in a variety of high-value products [9]. Natural polymers play a significant role in optimizing the extraction and stabilization of these compounds, enhancing their applicability and functionality in different sectors.

Isolation of these bioactive compounds from grapevine waste involves several extraction techniques, each tailored to preserve the integrity and bioactivity of specific compounds. Solvent extraction, one of the most commonly used methods, allows for the effective separation of polyphenols and flavonoids using solvents such as ethanol or methanol [125]. This method leverages the solubility of polyphenolic compounds, which dissolve readily in polar solvents, facilitating the separation of antioxidants from other plant materials. To enhance the sustainability of this process, green solvents, including water and ethanol, are increasingly being used to reduce the environmental impact associated with chemical extractions. Solvent extraction is often complemented by purification steps, such as filtration and evaporation, which concentrate the bioactive compounds and prepare them for downstream applications. However, the preservation of these compounds remains challenging due to their sensitivity to oxidation, necessitating the use of encapsulation techniques with natural polymers to maintain their stability and prolong their shelf life.

Advanced extraction techniques, including supercritical fluid extraction (SFE) and ultrasound-assisted extraction (UAE), have also gained popularity for isolating bioactive compounds from grapevine residues. Supercritical fluid extraction, which often uses carbon dioxide as the solvent under high pressure, is a non-toxic and highly efficient method for extracting heat-sensitive compounds, such as polyphenols and essential fatty acids [126]. This technique is particularly advantageous because it allows for precise control of temperature and pressure, minimizing the risk of degradation and preserving the bioactivity of the extracted compounds. Ultrasound-assisted extraction, meanwhile, uses high-frequency sound waves to disrupt cell walls, enhancing the release of intracellular compounds such as flavonoids and dietary fibers [127]. UAE is known for its reduced extraction time and lower solvent usage, which are beneficial from both an economic and environmental perspective. The application of natural polymers as encapsulating agents in these methods further enhances the stability of the isolated compounds, protecting them from oxidative and thermal degradation.

Once extracted, these bioactive compounds from grapevine waste can be applied across various industries, where they contribute not only to product functionality but also to sustainability. In agriculture, polyphenols and flavonoids from grape skins and seeds have shown potential as natural biopesticides due to their antioxidant and antimicrobial properties [128]. Encapsulated in natural polymers such as chitosan or alginate, these compounds can be delivered in controlled-release formulations that gradually release their bioactivity, providing prolonged protection against pathogens and pests [129]. These natural pesticide formulations offer a sustainable alternative to synthetic agrochemicals, reducing the environmental impact of vineyard management practices while enhancing crop resilience.

In the food industry, the antioxidants derived from grapevine waste are highly valued for their potential to enhance food preservation and nutritional quality [130]. Polyphenols, in particular, are sought after for their ability to prevent oxidation in food products, extending shelf life and reducing spoilage. By encapsulating these compounds in natural polymers like cellulose or alginate, food manufacturers can create additives that protect oils, meat products, and other perishable items from oxidative degradation [131]. Additionally, grape-derived dietary fibers, which are rich in indigestible polysaccharides, serve as functional ingredients in food formulations aimed at improving digestive health [132]. These fibers, when encapsulated, can be incorporated into dietary supplements or functional foods, where they promote a balanced gut microbiome and contribute to overall health. The encapsulation process also helps mask any potential bitterness from the polyphenols, making them more palatable for incorporation into food products.

In the cosmetics industry, the antioxidant and anti-inflammatory properties of polyphenols and flavonoids make them ideal for skincare and anti-aging products. Polyphenols from grape skins and seeds can neutralize free radicals, slowing down the visible signs of aging and protecting skin from environmental damage [133]. Encapsulated in biocompatible polymers like chitosan, these compounds can be delivered in sustained-release formulations, providing prolonged antioxidant effects that enhance the efficacy of cosmetic products [134]. Flavonoids, too, are beneficial in formulations that aim to reduce inflammation and improve skin elasticity, contributing to products that support skin health and vitality [135]. The ability of natural polymers to encapsulate and stabilize these sensitive compounds enables their incorporation into serums, creams, and lotions, where their bioactivity is preserved until application.

The pharmaceutical industry also benefits significantly from the bioactive compounds extracted from grapevine waste, as polyphenols and flavonoids have demonstrated a range of therapeutic properties, including cardioprotective, anticancer, and neuroprotective effects [136]. Resveratrol, for example, has garnered attention for its role in preventing oxidative stress and inflammation, making it a valuable component in supplements and pharmaceutical formulations aimed at promoting cardiovascular and cognitive health [137]. Encapsulation within natural polymers allows for targeted and controlled release, optimizing bioavailability and ensuring that these bioactive compounds reach their intended sites of action within the body [138]. Additionally, the use of biodegradable polymers for encapsulation minimizes the risk of toxicity and supports the development of safe and effective therapeutic products.

The value-added utilization of grapevine waste, facilitated by advanced extraction techniques and natural polymer encapsulation, exemplifies a sustainable approach that bridges waste management with the development of functional products. By transforming grapevine residues into valuable bioactive compounds, the grapevine industry not only addresses its own waste challenges but also contributes valuable raw materials to multiple sectors. The integration of natural polymers in the extraction, stabilization, and delivery of these compounds enhances their functionality and extends their application potential, fostering an innovative and circular approach to grapevine waste utilization. This model not only benefits the economic viability of the grapevine industry but also aligns with broader sustainability goals, reducing environmental impact and promoting responsible resource use across diverse industries.

### 5.3. Encapsulation and Stabilization of Bioactives Using Natural Polymers

The encapsulation and stabilization of bioactive compounds from grapevine waste using natural polymers represent an advanced approach to the value-added utilization of these abundant byproducts. Encapsulation allows for the efficient preservation and controlled release of bioactive compounds, such as polyphenols, flavonoids, and essential fatty acids, enhancing their functional potential across a variety of applications in the agriculture, food, pharmaceutical, and cosmetics industries. Natural polymer matrices, particularly those based on chitosan, alginate, and cellulose, offer numerous advantages for this purpose, including biocompatibility, biodegradability, and the ability to form versatile structures that support the stability and sustained release of encapsulated compounds.

The application of natural polymers for encapsulation is especially advantageous in controlled-release formulations. The gradual release of bioactives from these matrices enhances their stability and extends their functional lifespan, making them highly effective in applications that require prolonged activity, such as agricultural treatments or extended-release supplements. For instance, in agriculture, encapsulating grapevine-derived compounds like tannins and polyphenols in a chitosan or alginate matrix allows for their sustained release onto plants, providing continuous antioxidant and antimicrobial protection [139]. This reduces the need for frequent reapplication, thereby lowering labor costs and minimizing potential environmental impacts. The controlled release of these bioactives also enhances their efficacy, as compounds are not wasted through rapid degradation, but are instead available for plant uptake or pest deterrence over an extended period.

Stabilization through natural polymer encapsulation offers another crucial benefit by protecting sensitive compounds from environmental stressors. Polyphenols and flavonoids, while highly effective antioxidants, are susceptible to oxidation when exposed to air and light. Encapsulation in natural polymer matrices, such as chitosan or cellulose, shields these compounds from exposure, preserving their bioactivity. This is particularly valuable in food and nutraceutical applications, where the stability of antioxidants directly impacts product quality and efficacy. For example, grape seed polyphenols could be encapsulated within a biopolymer-based matrix can be incorporated into dietary supplements or functional foods, where the encapsulation not only prolongs shelf life but also ensures that the antioxidants remain active until they are consumed [140]. In pharmaceutical applications, this stabilization is equally essential, as it allows for the development of grapevine-derived therapeutics with improved bioavailability and consistent therapeutic effects over time.

Moreover, natural polymer-based encapsulation systems offer the flexibility to tailor the release and stabilization properties according to the specific needs of the target industry. By adjusting the polymer composition, cross-linking density, and encapsulation method, it is possible to customize the encapsulation process to suit the characteristics of the bioactive compounds and their intended application. In cosmetics, for instance, an alginate-based composite matrix might be used to encapsulate and slowly release grape-derived antioxidants over several hours, providing long-lasting skin protection and reducing oxidative damage [141,142]. This adaptability is particularly important in the grapevine industry, where the diversity of grapevine-derived compounds requires encapsulation systems that can cater to various physicochemical properties, including water solubility, thermal sensitivity, and molecular size.

In summary, the use of natural polymers such as chitosan, alginate, and cellulose for the encapsulation and stabilization of grapevine-derived bioactives provides significant advantages in enhancing the value of grapevine waste. Through controlled-release mechanisms and increased stability, these polymers allow for the efficient utilization of bioactive compounds in high-value applications, extending their functional life and enhancing their effectiveness. This approach not only supports sustainable waste management practices within the grapevine industry but also aligns with the growing demand for natural, biodegradable materials in product formulations across agriculture, food, cosmetics, and pharmaceuticals. As encapsulation technologies continue to advance, the integration of natural polymer-based matrices will undoubtedly play a pivotal role in transforming grapevine waste into a valuable resource, reinforcing sustainability and innovation in both viticulture and the broader field of bio-based materials.

### 5.4. Examples of Application in Different Industries

The utilization of grapevine waste in conjunction with natural polymers offers considerable potential across various industries, transforming these byproducts into high-value applications that capitalize on their bioactive content. Grapevine residues, which include grape skins, seeds, and stems, are rich in bioactive compounds such as polyphenols, flavonoids, and fibers. These compounds exhibit potent antioxidant, antimicrobial, and anti-inflammatory properties, making them desirable components in agriculture, food packaging, and pharmaceuticals. By embedding these bioactives within natural polymer matrices, industries can leverage their stability, controlled release, and functionality, creating products that enhance sustainability, promote health, and reduce waste.

In agriculture, the use of bioactive-enhanced polymer coatings derived from grapevine waste is an innovative solution for soil and crop treatments that simultaneously support plant health and pest control. Bioactive compounds from grape waste, such as polyphenols, tannins, and organic acids, demonstrate natural antimicrobial and antioxidant properties, making them effective against pathogens and pests when applied to crops. Encapsulating these compounds within natural polymers like chitosan or alginate enables controlled, gradual release onto the plant or soil, providing sustained protection against microbial infections and pests. For instance, a chitosan-based coating infused with plant polyphenols can be applied as a coating material on grapes to protect them from fungal pathogens in the post-harvest phase [143,144]. Chitosan’s innate antimicrobial properties further support this function, reducing the need for synthetic pesticides and fertilizers, which can be harmful to the environment and soil health. Additionally, these bioactive polymer coatings can be used in soil applications to improve soil health and nutrient availability, as grapevine polyphenols can stimulate beneficial soil microbes while deterring pathogenic species. This approach not only enhances crop resilience and reduces reliance on chemical treatments but also embodies an integrated method for recycling grapevine waste back into the vineyard, promoting a circular economy within viticulture.

In the food industry, the antioxidant and preservative properties of grapevine-derived bioactives are particularly beneficial when integrated into food packaging and preservation systems. Polyphenols from grape skins and seeds are powerful antioxidants that can significantly extend the shelf life of perishable food items by preventing lipid oxidation and microbial spoilage [145]. By embedding these polyphenols within natural polymer films such as cellulose or alginate, food packaging can be enhanced with antioxidant and antimicrobial capabilities, reducing the need for artificial preservatives. For example, biopolymer films (based on cellulose, alginate, chitosan, for example) infused with grape-derived flavonoids can be used to wrap fresh produce, meats, or dairy products, offering a protective barrier against oxygen and light that slows down spoilage [97]. This type of active packaging not only extends product freshness but also appeals to consumers who prefer natural, eco-friendly food preservation methods. Furthermore, grape-derived polyphenols encapsulated in alginate matrices can be integrated into edible coatings for fresh produce, forming a protective layer that prolongs shelf life without altering flavor or texture [97]. This application is particularly valuable in the preservation of highly perishable items like berries and salads, where the gradual release of antioxidants from the coating helps reduce microbial growth and oxidation, thus minimizing food waste and enhancing product appeal.

In the pharmaceutical sector, grape-derived polyphenols hold therapeutic potential for health supplements and cosmetics due to their well-documented antioxidant, anti-inflammatory, and cardioprotective effects. Resveratrol, for example, has attracted substantial interest for its potential to support cardiovascular health, reduce inflammation, and protect against oxidative stress, which contributes to aging and chronic diseases [146]. Encapsulation of resveratrol and other grape polyphenols within natural polymers such as chitosan or cellulose enhances their bioavailability and protects them from rapid degradation in the digestive tract, making these compounds more effective as health supplements [147]. By forming resveratrol-loaded chitosan nanoparticles, for example, pharmaceutical formulations can achieve targeted delivery to specific sites within the body, enhancing the compound’s therapeutic effects while minimizing degradation [148,149]. This approach enables the development of more effective natural health supplements that can provide consistent, sustained antioxidant benefits over time.

In cosmetics, the anti-aging and skin-protective properties of grape-derived polyphenols make them attractive ingredients in formulations aimed at improving skin health and appearance. When incorporated into polymer-based carriers, these polyphenols can be released gradually, providing prolonged antioxidant protection to the skin and neutralizing harmful free radicals generated by UV exposure and environmental pollutants [150]. For instance, resveratrol and other grape polyphenols can be encapsulated in alginate or alginate/chitosan microspheres which can be further used in moisturizing creams or serums, where their slow release enhances their stability and efficacy on the skin [151]. This controlled release not only extends the bioactivity of the antioxidants but also enhances user experience by providing long-lasting, visible benefits for skin elasticity, tone, and hydration. By harnessing grape-derived bioactives in stable polymeric forms, the cosmetic industry can offer products that promote anti-aging and skin protection using naturally derived, sustainable ingredients.

Overall, the integration of natural polymers with grapevine-derived bioactives across these industries illustrates a versatile and sustainable approach to waste utilization. By using natural polymer matrices to encapsulate and stabilize these compounds, industries can harness the functional benefits of grapevine waste in applications that meet the growing consumer demand for environmentally friendly, health-promoting products. The agricultural sector benefits from the sustainable crop protection offered by bioactive polymer coatings, reducing dependence on synthetic pesticides. In the food industry, antioxidant-enhanced packaging extends product shelf life and reduces food waste, contributing to both environmental and economic sustainability. Meanwhile, the pharmaceutical and cosmetic industries gain access to high-value bioactives that are protected within natural polymer carriers, facilitating their use in therapeutic and skincare applications with enhanced bioavailability and controlled release. Together, these applications not only reduce grapevine waste but also contribute to a more circular, sustainable model within the grapevine industry, highlighting the transformative potential of natural polymers in adding value across multiple sectors.

## 6. Case Studies of Natural Polymer Applications in the Grapevine Industry

### 6.1. Real-World Examples and Research Studies

In recent years, research into the application of natural polymers within the grapevine industry has demonstrated substantial promise, with multiple studies underscoring their effectiveness in areas such as plant protection, crop yield improvement, and post-harvest preservation. These case studies not only highlight the potential of natural polymers as sustainable tools in viticulture but also provide valuable insights into their mechanisms and practical benefits in real-world settings. By examining both laboratory and field research, as well as applied practices, it becomes clear that natural polymers offer a multifaceted approach to enhancing grapevine resilience, maximizing yield, and extending product lifespan, which collectively address critical needs in the grapevine industry.

One of the most important and studied areas of research is focused on the development of chitosan-based coatings for grapevine post-harvest diseases management. Some of the relevant findings on this topic are presented in Table 2.

As previously presented, chitosan has shown strong antimicrobial and antifungal activity, making it highly effective in protecting grapevines against pathogens such as *Botrytis cinerea*, the causative agent of gray mold. In several experimental trials (presented in Table 2), chitosan-based treatments applied to grapevines resulted in reduced disease incidence and severity. More than that, the treatments, usually applied as edible coatings, led to an increase in the quality parameters of the grapes, while prolonging their shelf-life.

Similar approaches were also considered for other natural polymers, although not as encountered in the literature. For example, Liu et al. [95] proposed an edible coating composed of sodium alginate containing an inclusion complex of *Magnoliae* essential oil and β-cyclodextrin to extend the shelf life of Kyoho grapes, at room temperature. The treatment led to a significant reduction of the total color difference, decay rate, weight loss rate, enzyme activity, and total number of bacterial colonies, while also slowing the decline in hardness and total soluble solids, when compared with untreated samples.

Beyond the post-harvest approach, the natural polymers have been also (usually by foliar application) applied to improve the resilience of grapevine against different pathogens in field trials, at different vegetative stages. Different chitosan-based formulations were applied in multiple-years studies to prevent grapevine downy mildew [79,83,166,167,168,169,170,171,172], powdery mildew [79,173], grapevine leaf spot [170], fungi and bacteria related to trunk diseases [174], grey mold [175], *Botryosphaeriaceae* fungal species [176], or as nematicide agent, as demonstrated by Abdel-Sattar et al. [177] against the root-knot nematode *Meloidogyne incognita*. The use of chitosan (in different formulations, with or without the addition of other anti-pathogen agents) led not only to an increase in pathogen resistance, but was also proven to act as biostimulants, increasing the phenolic acids, terpenoids, anthocyanins, and other important secondary metabolites [169,178,179,180,181,182,183,184,185,186] in grapes, while also being able to improve micronutrient up-take [187]. More important, the influence is also visible on the wine produced, improving its quality [80,178,188,189].

Chitosan-based formulations were also proven to improve grapevine resilience to abiotic stressors, particularly drought and heat stress, which are increasingly prevalent in regions impacted by climate change [190], but also as a protective agent of grapevine under salinity stress [191]. Applied on grapevine, these types of composites exhibit a great potential for the development of a more sustainable viticulture [65].

On the other hand, chitosan can also be used as a vehicle for the incorporation of valuable biological active compounds resulted from viticulture practices, such as resveratrol, which could be further used for increasing the shelf life of other products, such as mango [192], or for incorporating wine lees extracts [193] or grape seed oil [194], thus allowing the superior valorization of different by-products.

Alginate has also been employed in experimental settings to encapsulate grape pomace extract, thus creating a viable vehicle for the oral administration of the phenol-rich extract [49,195]. Resveratrol was used to develop a sodium alginate-based edible coating for increasing the shelf life of rainbow trout fillet during refrigerated storage, at different concentrations by Bazargani-Gilani [196]. According to the presented results, the treatments led to a significant decrease in total viable counts, psychrotrophic bacteria, *Enterobacteriaceae*, *Pseudomonas* spp., lactic acid bacteria, and yeasts–molds compared to the control in the storage period. In a similar approach, Capar [197] developed a biodegradable edible film of sodium alginate in which was incorporated *Vitis vinifera* leaf extract and quercetin, thus developing antioxidative and antimicrobial alternative for food packaging.

Together, these case studies highlight the considerable advantages of natural polymers in supporting the grapevine industry’s shift toward sustainable practices. The successful application of natural polymers in disease management, stress tolerance, and post-harvest preservation underscores their versatility and effectiveness as alternatives to conventional chemical treatments. By integrating natural polymers into grapevine production and post-harvest handling, viticulturists can enhance crop resilience, improve yield, and reduce waste, ultimately aligning with the industry’s goals for environmental stewardship and economic viability. Further research and field trials continue to refine these applications, with ongoing efforts to optimize polymer formulations for different grape varieties, climates, and industry needs. As the body of evidence grows, the adoption of natural polymer-based solutions is likely to expand, establishing them as essential components in the sustainable advancement of the grapevine industry.

### 6.2. Implementation Challenges and Lessons Learned

Implementing natural polymers in the grapevine industry, while promising, has encountered several challenges that require strategic solutions to achieve scalable and practical applications. In transitioning from research to real-world applications, vineyards face a unique set of challenges across regulatory, practical, and economic dimensions. These difficulties highlight both the complexity of integrating novel biotechnologies into agricultural systems and the need for continued optimization of polymer formulations to meet industry standards and field requirements effectively.

One significant challenge in implementing natural polymer-based applications in viticulture arises from regulatory frameworks that govern the use of new agricultural treatments. In many regions, the introduction of biopolymer formulations on crops is subject to rigorous scrutiny to ensure that they are safe, environmentally friendly, and pose no adverse effects to human health. Regulatory bodies often require extensive documentation demonstrating the biocompatibility, biodegradability, and non-toxicity of these polymers, particularly when they are applied directly to edible crops like grapes. While natural polymers are generally considered safe, variations in purity, production methods, and formulation additives can complicate regulatory approval processes. For example, certain chitosan formulations may require proof of negligible allergenicity, as they are derived from shellfish, which is a common allergen [198]. Meeting these regulatory standards can be costly and time-consuming, which may deter some producers from adopting polymer-based treatments unless they are provided with clear, standardized guidelines and robust evidence of efficacy.

Beyond regulatory hurdles, practical challenges in field applications have surfaced, especially concerning the stability, application techniques, and performance of these biopolymers under variable environmental conditions. Vineyards operate in diverse climates with different temperature ranges, humidity levels, and seasonal variations, which affect the performance of natural polymer coatings and treatments. Chitosan, for instance, may perform effectively under certain conditions but degrade or lose its antimicrobial potency in high-temperature or high-humidity environments [199]. Field trials have shown that optimizing polymer formulations for specific environmental conditions is essential to achieve consistent results. This often requires the addition of stabilizing agents or the development of composite coatings that combine multiple polymers, such as chitosan with cellulose or alginate, to enhance robustness. Lessons from field studies suggest that polymer formulations must be tailored not only to the crop’s needs but also to the vineyard’s unique environmental parameters, and achieving this balance requires extensive preliminary testing.

Another critical practical issue is the method of application. Many natural polymers, especially those used in coatings or foliar sprays, require even and controlled distribution to ensure effective coverage of grapevine leaves, stems, or fruit. In large vineyard operations, this application can be logistically challenging, especially if the formulation needs to be reapplied periodically to maintain efficacy against pests or environmental stressors. Traditional spraying equipment may need to be adapted to handle the viscosity and consistency of certain polymer-based solutions, which can differ from conventional chemical treatments. In several case studies, field operators noted that some polymer coatings tended to clog standard spray nozzles, leading to inconsistent application and inefficiencies. This has driven research into the development of more efficient delivery systems, such as microencapsulation techniques or modified spray equipment designed specifically for polymer-based solutions. Lessons from these cases emphasize that the effectiveness of natural polymers in real-world vineyard settings depends not only on the formulation’s chemistry but also on the compatibility of delivery methods with existing agricultural practices.

Economic factors also play a significant role in the adoption and success of natural polymer applications in viticulture. While natural polymers like chitosan and alginate are generally cost-effective in small-scale or laboratory settings, scaling production for extensive vineyard use can elevate costs substantially. The cost of raw materials, extraction processes, and formulation development must be balanced with the financial benefits they offer, such as reduced reliance on synthetic chemicals, improved crop resilience, or extended post-harvest shelf life. However, vineyards operating on tight margins may find it challenging to justify the initial investment required for transitioning to these biopolymer-based treatments, particularly if they have yet to yield clearly quantifiable economic returns in the short term. Case studies underscore the importance of establishing clear economic models and cost-benefit analyses that demonstrate the value of polymer applications over time. For instance, data showing reduced crop losses due to pest resistance or lower post-harvest spoilage rates can make a compelling case for adoption, especially for high-value grape varieties where even marginal improvements in yield can translate into substantial economic gains.

When considering small-scale vineyards, where efficiency and resource optimization are paramount, natural polymers also offers other solutions, such as natural polymers-based mulches. These mulches suppress weed growth, regulate soil temperature, and conserve moisture. While the initial cost of natural mulches tends to be higher than synthetic options, their ability to biodegrade in situ eliminates the need for removal and disposal, significantly reducing labor costs over time. Furthermore, by gradually decomposing, these materials enrich the soil with organic matter, potentially decreasing the reliance on external fertilizers and enhancing long-term vineyard productivity. For small-scale operations, where manual labor often represents a significant portion of expenses, these savings can offset the higher upfront investment [200].

Regarding the application of protective coatings for vines and fruits, although the cost of these coatings may exceed that of conventional chemical treatments, they often provide added value by catering to the growing consumer demand for organic and eco-friendly produce. This demand can translate into premium pricing for the vineyard’s products, helping to justify the initial investment in natural polymer-based solutions [201].

Overall, the cost-effectiveness of natural polymers in small-scale vineyards hinges on their ability to reduce labor and disposal costs, improve soil and plant health, and align with market trends favoring sustainable practices. While the initial expenses may be higher, the long-term economic and environmental benefits often outweigh these costs. For small-scale vintners, especially those cultivating premium or organic labels, natural polymer-based solutions represent not only an environmentally responsible choice but also a strategic investment in the future viability of their operations [202].

Another lesson learned is the value of localized production of biopolymers, where vineyards can collaborate with regional processors to source polymers like chitosan or cellulose from locally available waste materials, such as crustacean shells or agricultural residues. This localized production model not only reduces costs associated with transportation and imports but also supports regional economies and promotes sustainability by repurposing waste.

The adoption of natural polymers across agricultural and viticultural applications is influenced by regional and global trends, driven by environmental policies, market demands, and advancements in material technology. These trends provide critical context for understanding the viability and potential of natural polymer solutions, particularly in small-scale vineyards.

Globally, there is a pronounced shift towards sustainability in agriculture, motivated by increasing awareness of the environmental damage caused by synthetic polymers. Governments and international organizations have begun to implement stringent regulations targeting single-use plastics and non-biodegradable materials [203]. For instance, the European Union’s directive on reducing plastic waste has incentivized the adoption of biodegradable alternatives, including natural polymers, across various sectors [204]. Similarly, countries like China, Japan, USA, or Canada have introduced subsidies and tax benefits to encourage the use of bio-based materials in farming, including vineyards [205].

In regions such as Europe, where viticulture is a significant industry, the adoption of natural polymer solutions is particularly advanced. European vineyards, often operating under organic or sustainable certifications, have embraced natural polymer-based solutions to align with regulatory frameworks and meet consumer expectations [7]. These trends are supported by a robust research and development ecosystem, with institutions actively exploring cost-effective natural polymer formulations tailored to regional needs.

In North America, the adoption of natural polymers in viticulture is growing steadily, bolstered by the increasing consumer demand for organic and eco-friendly wines. California, a major wine-producing region, has seen a surge in the use of sustainable agricultural practices, including biodegradable vine ties and soil covers. Small-scale vineyards in this region often leverage natural polymers as part of broader sustainability narratives to differentiate their products in a competitive market [206].

Meanwhile, in emerging wine-producing regions such as South America, Southeast Asia, and Africa, the adoption of natural polymers is still in its nascent stages. Economic constraints and limited access to advanced materials have hindered widespread implementation [207]. However, international collaborations and funding from global sustainability initiatives are beginning to facilitate the introduction of affordable, locally sourced natural polymer solutions.

On a global scale, market dynamics are also shaping the adoption of natural polymers. The rising costs of petrochemical-derived materials, coupled with volatile oil prices, have made bio-based alternatives more attractive. Additionally, advancements in material science, or enhanced processing techniques for natural fibers, have reduced the cost disparity between natural and synthetic polymers, further driving their adoption [60].

Overall, the adoption of natural polymers is strongly aligned with global sustainability goals and consumer preferences. Regional variations, however, depend on economic, environmental, and regulatory factors. These trends suggest that while adoption is more advanced in developed markets with strong sustainability policies, emerging regions hold significant potential for growth, particularly with increased investment in local material development and infrastructure [208]. For small-scale vineyards, aligning with these trends not only enhances environmental stewardship but also provides a competitive edge in increasingly eco-conscious markets [209].

In summary, while natural polymers offer transformative benefits for the grapevine industry, their implementation in vineyards requires overcoming several challenges. The regulatory landscape demands rigorous safety and environmental assessments, while practical obstacles in field application and economic feasibility present additional layers of complexity. Insights from case studies suggest that the successful integration of natural polymers in viticulture hinges on comprehensive formulation optimization tailored to specific environmental conditions, effective and compatible delivery systems, and economically sustainable models of production and use. By addressing these factors, the grapevine industry can unlock the full potential of natural polymers, paving the way for resilient, sustainable, and economically viable grape production that meets both industry and environmental objectives.

## 7. Future Perspectives and Research Directions

### 7.1. Innovation Opportunities

Looking forward, the use of natural polymers in the grapevine industry offers a vast frontier for innovation, especially as emerging technologies like nanotechnology, precision agriculture, and advanced polymer science converge. Future research and development in this area can fundamentally reshape the grapevine industry by creating more effective, sustainable, and tailored solutions that address specific challenges within vineyards. The integration of natural polymers with nanotechnology, for instance, presents compelling opportunities to enhance the performance of biopolymer treatments and coatings, while precision agriculture can enable their optimized application at precise locations and doses. Additionally, exploring new sources of biopolymers and developing novel polymer blends can lead to materials with specialized properties that improve plant protection, environmental resilience, and waste management in viticulture.

Nanotechnology has significant potential to augment the effectiveness of natural polymer applications in vineyards. By engineering natural polymers at the nanoscale, researchers can improve their delivery, stability, and targeted activity. For example, nanoscale particles of chitosan can increase its adhesion to grapevine surfaces and facilitate controlled release of bioactive compounds. Additionally, incorporating essential oils or antimicrobial agents into nanoparticles of chitosan or alginate allows for highly targeted pathogen control, as these nanoparticles can be engineered to release their active components upon specific triggers, such as humidity or pH changes in the plant environment. By leveraging such advancements, nanotechnology can enable natural polymers to act not only as carriers for bioactives but also as responsive systems that adjust their activity based on real-time environmental conditions, effectively creating a smart treatment system.

Precision agriculture further supports the effectiveness of natural polymer applications by enabling precise, data-driven decision-making for applying treatments in vineyards. With the increasing use of drones, remote sensing, and machine learning in agriculture, natural polymer treatments can be deployed with exceptional spatial and temporal accuracy, targeting only areas or vines that require intervention. This level of precision minimizes waste and ensures that polymers are applied in the most efficient way possible, thereby reducing the amount of material needed and decreasing overall costs. Precision agriculture can also help monitor the effects of natural polymer treatments over time, using sensors to detect changes in plant health, stress levels, or pathogen presence, and thereby enabling adjustments in treatment regimens based on live data. The potential to integrate natural polymer applications with real-time monitoring and predictive analytics opens up pathways for more efficient, sustainable, and responsive viticulture practices, where interventions are guided by plant-specific needs and optimized for each vineyard’s unique environment.

In parallel with technological integration, exploration of novel natural polymers and the development of polymer blends offer additional avenues for advancing the role of biopolymers in viticulture. While chitosan, alginate, and cellulose are well-studied and widely utilized, other biopolymers such as pectin, starch derivatives, and gums have unique properties that could be beneficial in vineyard applications. Pectin, for example, is known for its gel-forming ability [210] and could be particularly effective as a coating that provides a humid microenvironment around grapevine roots or leaves during drought conditions. Starch-based polymers, often regarded for their low cost and availability [211], can also be chemically modified to improve their water retention and adhesion properties, making them suitable for applications in regions where water stress is a critical issue. Blending various natural polymers offers the potential to create composite materials that capitalize on the strengths of each component.

To realize the full potential of these innovations, further research is needed to address key technical and operational challenges. For instance, scaling up production of novel polymers and polymer blends from laboratory settings to industrial-scale quantities must be achieved without compromising quality, consistency, or cost-effectiveness. Comprehensive field trials are essential to test new formulations under diverse environmental conditions and validate their effectiveness in real vineyard operations. Such trials must account for varying temperatures, humidity levels, and pest pressures, as well as the interactions between different biopolymers and active compounds used in combination treatments. Additionally, research should focus on understanding the long-term environmental impacts of applying natural polymer treatments at scale, including their effects on soil health, microbial ecosystems, and vineyard biodiversity. Addressing these questions through robust experimentation and interdisciplinary collaboration will be essential to advancing natural polymers from niche applications to mainstream solutions within the grapevine industry.

### 7.2. Sustainability and Economic Viability

The future of natural polymer applications in the grapevine industry lies strongly within the framework of a circular economy, where resource efficiency and waste valorization play central roles. Shifting toward a circular economy model represents an opportunity to transform the traditional vineyard management system into one that minimizes waste, maximizes resource utilization, and supports environmental and economic sustainability. In this model, natural polymers and grapevine by-products are integrated into production processes to create value from waste materials, promoting a closed-loop system. The effective reuse of grapevine residues and the strategic application of natural polymers both for plant protection and as carriers for bioactive compounds align seamlessly with circular principles, underscoring how biotechnological innovation can support more sustainable agricultural practices.

Within a circular economy approach, the grapevine industry can benefit from waste valorization by turning by-products such as grape skins, seeds, and stems into sources of valuable bioactive compounds, which can then be encapsulated or stabilized using natural polymers. This transformation of waste into value-added products allows vineyards to reduce environmental impact by decreasing organic waste output and, at the same time, tapping into new revenue streams. For example, grape seeds and skins are rich in polyphenols, flavonoids, and fibers, which can be extracted and repurposed in various industries, including food, cosmetics, and nutraceuticals. When these bioactive compounds are encapsulated in natural polymer matrices like chitosan, alginate, or cellulose, they gain improved stability and controlled release properties, which enhance their efficacy and broaden their application potential. By utilizing these residues, vineyards can lessen the need for synthetic chemicals in the field, reduce dependency on external inputs, and adopt a more integrated approach that aligns agricultural productivity with ecological health.

The circular model’s impact is particularly evident when examining the economic and environmental benefits associated with natural polymer applications on a large scale. By substituting conventional synthetic agrochemicals with biodegradable natural polymers, vineyards can reduce the ecological footprint associated with conventional pest and pathogen management practices. Natural polymers, due to their non-toxic, biodegradable nature, offer a safe alternative that minimizes soil and water contamination risks, fostering healthier vineyard ecosystems and reducing long-term environmental liabilities. Moreover, biopolymers used for crop protection, such as chitosan, have demonstrated a broad spectrum of benefits, from enhancing plant resistance to pathogens to improving resilience against abiotic stressors. By using such treatments, vineyards can achieve more sustainable crop management practices that potentially lead to lower chemical costs and, in the long term, fewer remediation expenses for environmental damage.

In considering the cost-benefit dynamics of natural polymers in large-scale applications, it is clear that initial investments in natural polymer-based treatments may be higher than traditional synthetic counterparts, largely due to material costs and, in some cases, the specialized application methods required. However, these costs are often offset by the multiple benefits natural polymers provide, especially in terms of reduced input needs, crop protection efficacy, and potential yield improvements. Case studies have illustrated how natural polymers, such as chitosan-based coatings, not only act as effective antimicrobial barriers but also promote plant growth and enhance crop yield through biostimulant properties. For high-value grape varieties, even marginal yield improvements can translate into significant economic returns, which make the upfront investment in natural polymers financially justifiable over time.

Cost-effectiveness can also be improved by optimizing production and sourcing. As research advances, methods for producing natural polymers from readily available agricultural or marine sources have become more efficient. The utilization of regional resources for biopolymer production, such as chitosan from local shellfish waste or alginate from local seaweed, can significantly reduce transportation costs and improve economic sustainability for vineyard operations. By sourcing biopolymers from waste materials, particularly agricultural residues, vineyards can both lower input costs and participate actively in circular economy initiatives, further promoting sustainability. Additionally, incorporating grapevine waste into the production of cellulose-based films and coatings, which can then be reapplied to vineyards, exemplifies a truly circular model where waste becomes a vital input for vineyard health and productivity.

Future research should also focus on improving the scalability of natural polymer production and application processes to reduce costs. Investigations into durable, efficient application systems, such as automated sprayers designed to handle polymer-based treatments, will further enhance economic feasibility. By ensuring that large-scale applications are efficient and compatible with existing vineyard practices, the cost of natural polymer usage can be minimized, allowing vineyards of various sizes to benefit from this approach.

From a long-term perspective, the adoption of natural polymers also opens up new market opportunities that contribute to economic viability. In markets increasingly driven by consumer preference for sustainability, organically cultivated and treated grapes that utilize biodegradable, non-toxic polymer treatments can command premium prices. Wines or grape products marketed with certifications that highlight environmentally friendly practices and reduced synthetic chemical usage can leverage this consumer demand. Natural polymers thus support not only sustainable viticulture practices but also create branding opportunities that align with current market trends favoring ecological responsibility. Consequently, vineyards that adopt natural polymers could benefit from both direct cost savings in chemical inputs and higher revenues from value-added branding.

Ultimately, the integration of natural polymers into the grapevine industry as part of a circular economy model represents a viable strategy for aligning viticulture practices with the dual goals of sustainability and economic resilience. By valorizing grapevine waste, reducing environmental impact, and enhancing crop resilience, the use of natural polymers contributes to a more resource-efficient, sustainable agricultural system. Continued research on optimizing these polymers for large-scale use and improving cost-efficiency in application methods will further accelerate the industry’s transition towards this sustainable future, positioning natural polymers not only as effective tools for viticulture but as integral components in the evolution of a more circular, responsible grapevine industry

### 7.3. Future Research Opportunities

In considering the future of natural polymer applications in the grapevine industry, further research is essential to fully understand and harness the potential of these materials, ensuring they deliver optimal benefits for viticulture while meeting environmental and economic demands. A primary research direction involves investigating the long-term impacts of natural polymer applications on both soil health and plant physiology. Although natural polymers are biodegradable and generally regarded as safe, detailed studies are needed to assess their persistence, breakdown products, and any cumulative effects they might have over multiple seasons of application. Long-term field trials that monitor soil microbial communities, nutrient availability, and soil structure after repeated exposure to natural polymers would provide invaluable insights. Additionally, understanding how these polymers interact with plant roots, nutrient uptake, and grapevine physiology under various environmental conditions can help optimize their use in vineyard settings, ensuring that short-term benefits do not compromise long-term soil and plant health.

Another crucial area for research lies in refining polymer formulations to maximize their effectiveness while balancing costs and minimizing environmental impacts. Different natural polymers, such as chitosan, alginate, and cellulose, each bring unique properties to their applications, and optimizing these properties to suit specific vineyard needs remains a priority. For instance, chitosan’s antimicrobial properties can be highly effective against pathogens, but its efficacy and longevity may be improved through cross-linking with other biodegradable materials or by combining it with substances that enhance plant defenses. The development of tailored formulations that incorporate additives to improve stability, enhance bioactivity, or adjust release rates of active compounds could greatly enhance their field efficacy. Similarly, the integration of nanotechnology in polymer formulations, such as creating nanoscale carriers for more precise release of active compounds, could allow for more targeted application and reduce the total amount of polymer required, improving cost-efficiency and environmental compatibility.

The cost of production and application of natural polymers is another critical factor that warrants attention. Research aimed at streamlining the production process of biopolymers to make them more economically viable on a commercial scale is essential. Cost reductions can be achieved by exploring alternative sources for polymer production, such as waste-derived feedstocks. For example, chitosan can be derived from seafood waste, and finding efficient ways to use this or other agricultural residues could reduce the cost of raw materials. Research should also explore techniques like scalable spray drying or cost-effective electrospinning to produce polymers in a manner compatible with large-scale application in vineyards. Through optimization, these formulations can become more accessible to growers, enabling broader adoption and making natural polymers a practical choice even for smaller or resource-limited vineyards.

Furthermore, the potential of grapevine waste applications beyond polyphenols represents a promising yet underexplored area of study. Grape residues, including seeds, skins, and stems, contain a rich array of bioactive compounds beyond polyphenols, such as stilbenes, flavonoids, tannins, and dietary fibers, each with distinct health-promoting or functional properties. These compounds hold considerable promise not only in traditional areas like the food and cosmetic industries but also in fields such as agriculture, where they could be used to develop new biostimulants, or in medicine for potential therapeutic applications. For instance, stilbenes, including resveratrol, are known for their antioxidant properties and may hold untapped potential in the formulation of natural health supplements or as additives in crop treatments to support plant resilience. Research dedicated to identifying and isolating these compounds efficiently and assessing their compatibility with natural polymer encapsulation or stabilization could open up novel avenues for grapevine waste utilization. By broadening the range of bioactive compounds extracted from grapevine residues, the industry can diversify its value-added products, creating new markets and economic opportunities.

## 8. Conclusions

The present review highlights the transformative potential of natural polymers within the grapevine industry, focusing on their dual capacity to both protect grapevines and drive sustainable, value-added utilization of vineyard waste. As the industry faces significant challenges related to environmental stressors, pathogens, and sustainability, natural polymers offer a biologically compatible, environmentally friendly alternative to synthetic agrochemicals, reinforcing crop resilience while simultaneously aligning with circular economy principles. Through biodegradable, non-toxic formulations, these polymers such as chitosan, alginate, and cellulose provide multiple modes of action, from antimicrobial and antifungal effects to physical barriers that help plants withstand temperature fluctuations and drought. By forming protective coatings on plants, natural polymers enhance grapevine resistance against pathogens, insects, and adverse weather, thereby contributing to stable yields, reduced crop losses, and improved fruit quality. These natural polymer-based treatments not only reduce the need for conventional pesticides and fertilizers but also promote more sustainable agricultural practices that minimize chemical inputs and safeguard soil and water health.

Natural polymers can serve as effective agents for both grapevine protection and waste utilization, combining ecological benefits with economic advantages. By advancing research on polymer formulations, exploring new bioactive extractions, and assessing long-term impacts, the grapevine industry can optimize the use of natural polymers and further reinforce the sustainability and resilience of viticulture.

In conclusion, the path forward for the grapevine industry lies in a deep commitment to sustainable practices, propelled by the transformative potential of biopolymers and a unified approach to research and development. This study calls for an industry-wide movement toward embracing these natural, regenerative solutions, which have the capacity not only to protect grapevines and enhance production but also to support broader environmental objectives that resonate with today’s consumers and stakeholders. As the industry embraces these sustainable solutions, it is essential that researchers, policymakers, and industry leaders work hand in hand to develop, implement, and promote biopolymer-based innovations that will underpin a more resilient and economically viable future for viticulture. In doing so, the grapevine industry can emerge as a model for sustainable agriculture, demonstrating how traditional practices can be harmonized with modern scientific advancements to create an industry that is both productive and ecologically responsible.

## Figures and Tables

**Table 1:** Overview of natural polymers selected for the study.

Natural Polymer/ Properties	Chitosan	Alginate	Cellulose
Macromolecular classification	Polysaccharide	Polysaccharide	Polysaccharide
Main source	Animals	Algae	Plants
Characteristics	The only polycation in nature, charge density depends on acetylation degree and media pH, biocompatible and biodegradable. Tunable solubility, depending on acetylation degree and molecular weight [37].	Biocompatible and biodegradable, more stable as powder, insoluble in ether, chloroform, alcohol, and hydroalcoholic solutions (>30% alcohol); at low pH forms a gel of alginic acid. Tunable physical characteristics (tensile strength, elasticity, and viscosity) and gelling rate, dependent on the molecular weight [38]	Low density, lack of abrasiveness, lack of toxicity, biocompatible, biodegradable, relatively high tensile/flexural modulus [39]
Applications	Multiple applications, including pharmaceutical, biomedical, cosmetics and food industries [37]	Commonly used in different sectors, including cosmetic, textiles, wastewater treatment, wound dressing, development of smart materials [38]	Food industry, packaging, pharmaceutical, medical, waste water treatments [39].

**Table 2:** Application of chitosan as a post-harvest treatment on different cultivars (cv.). Studies are presented in chronological order.

Coating Formulation	Grape Cultivar	Main Findings	Ref.
Grapes dipped in chitosan (100,000–300,000 MW) solution at 1, 1.5 or 2% (with Tween 20 at 1 mL/L) for 10 s.	“El-Bayadi”	Decreased decay after storage (2.95–4.42%) compared to control (16.25%). Weight loss lower at 1% chitosan (0.39%), compared with other concentrations and control (1.66). Minimum changes in total soluble solids, acidity and pH of berries, not affected by chitosan treatments. Higher firmness than control recorded. Chitosan at 1%, maintained higher total phenols and flavonoids concentration than control. Total phenols concentration decreased as chitosan rate increased, in contrast to total flavonoids. Vitamin C increased after storage than initial, not affected by chitosan treatments. Peroxidase-higher activity after storage, in all treatments, higher in chitosan treatments than control, increasing as chitosan concentration increased. Polyphenoloxidase-lower activity after storage in control compared to initial. Chitosan treatments maintained higher polygalacturonase and xylanase activities than control, increasing with chitosan concentration. Chitosan treatments increased antioxidant capacity (DPPH) compared with control.	[152]
Grapes immersed in chitosan/polyvinyl alcohol (CS/PVA) blended with ascorbic acid (AA) at different concentration (0, 2.8, 5.6 and 8.2 mM) for 5 min.	“Superior Seedless”	Highest concentration treatment reduced water loss, inhibited cell wall degrading enzymes (cellulase, polygalacturonase, xylanase), reducing the cell membrane permeability and decreasing rachis browning by inhibiting polyphenol oxidase enzyme, maintained total polyphenols and total flavonoids, increased antioxidant activity	[153]
Grapes immersed in chitosan/polyvinyl alcohol (CS/PVA), mixed with salicylic acid (SA) at various doses (0, 1, and 2 mM) for 5 min.	“Thompson seedless”	CS/PVA-SA 2 mM reduced water loss and berry shattering, postponed the change in berry color hue angle. The cell wall enzyme activities (CEL, PG, and XYL) were inhibited compared with different treatments.	[154]
Chitosan nanoparticles prepared by ionic gelation. To develop the coatings, chitosan solubilized in 1% acetic acid (20 g/L) and nanoparticles were added. Grapes immersed in solution for 3 min.	*Vitis labrusca* L.	The treatment delayed the ripening process, decreased weight loss, soluble solids and reducing sugar contents, increased moisture retention and preservation of the titratable acidity values and sensory characteristics	[155]
Grapes dipped in 1%/2% chitosan coatings for 10 min.	“Sagrantino”	Chitosan coatings delayed water, no difference in berry color/peel resistance during partial dehydration (up to 30% mass loss). Reducing sugar content increased from 275 g/L (harvest), to 445/428 g/L (2%, respectively 1% chitosan). Malic acid, and total acidity increased. Highest total polyphenol registered at 1% chitosan treatment. Treatments enhanced activity of antioxidant enzymes, superoxide dismutase and ascorbate peroxidase, during partial dehydration process, inhibited polyphenoloxidase and lipoxygenase activity, preventing polyphenol loss and avoiding membrane oxidation	[156]
Chitosan applied at 0.5/1%	“Red Globe”	Chitosan was effective as biocontrol agent against *A. alternata* (incidence rates similar to traditional control-SO_2_), being more effective in reducing the incidence of *A. alternata* when applied after the *Alternaria* inoculum, suggesting that chitosan represents a viable curative treatment.	[157]
Grapes immersed in 1.5% chitosan solution and 2% acetic acid solution for 1 min	“Kyoho” and “Shine Muscat”	Chitosan treatment inhibited the damage of *Botrytis cinerea* to grapes, destroyed the mycelium of *B. cinerea,* increased the antioxidant enzyme activities of grapes. The treatment led to significant differences in the two cultivars, including secondary metabolites level (epigallocatechin gallate, catechin, resveratrol etc.).	[158]
Grapes dipped in low molecular weight chitosan solution (1%, *w/v*) prepared in acetic acid	“Italia”	The CO_2_-modified atmosphere in combination with chitosan preserved quality, sensorial parameters, and delayed grapes’ decay. Addition of chitosan treatment to modified atmosphere packing MAP creates a natural film on rachis and berries surface, protecting the fruit against pathogen infection, reducing respiration rate, and decreasing decay.	[159]
Grapes treated with 1.0% chitosan (150 kDa)	“Kyoho” and “Shine Muscat”	Chitosan inhibited *B. cinerea* growth, increased phenolic compounds and cell wall composition, modulated oxidative stress and induced jasmonic acid (JA) production in ripened fruits. 224 and 171 proteins were upregulated 1.5-fold by chitosan in Kyoho and Shine-Muscat grape, respectively. Topless-related protein 3 (TPR3) were identified and interacted with histone deacetylase 19 (HDAC19) and negatively regulated by JA and chitosan	[160]
Grapes dipped in chitosan and Gum ghatti formulated at different concentrations of each component (0, 0.5, 1.0%) for 5 min.	“Rishbaba”	Coatings positively influenced berry softening, discoloration, drop and rachis browning (coating with 1% chitosan and 1% Gum ghatti changes reduced parameters 2.5 to 6 times compared with control). The composite treatment improved berry texture and sensory scores (17 scores higher than control). Significant differences in pH and total soluble solids between coated and uncoated grapes, delayed changes in ascorbic acid, membrane stability of grapes after 20 days of storage, had positive effects on the antioxidant enzyme activities that preserved the enzymatic browning of fruits_._	[161]
Grapes immersed in chitosan oligomers (COS) complexes with bacterial secondary metabolites (COS–*Streptomyces. lavendofoliae* DSM 40217, COS–*S. rochei* DSM 41729) for 5 min.	“Red Globe” and “Timpson”	Treatments maintained the turgor of the grapes and delay the appearance of the pathogen by 10−15 days at concentrations in the 750−1000 µg/mL range. COS–*S. rochei* DSM 41729 secondary metabolites conjugate complex (750 μg/mL) fully inhibited the growth of *B. cinerea* on cv. “Timpson” and cv. “Red Globe” grapes for 15 days. COS–*S. lavendofoliae* DSM 40217 secondary metabolites conjugate complex (1000 μg/mL), full protected cv. “Timpson” berries, registering a 20% incidence for “Red Globe” cv. grapes after 15 days.	[162]
Grapes dipped in 0.5% chitosan (medium molecular weight), 200 mg/L thymol and 0.5% chitosan + 200 mg/L for 2 min.	“Bidaneh Ghermez”	Treatments led to higher levels of firmness, anthocyanin, antioxidant activity, and sensory attributes, increased the activity of guaiacol peroxidase and catalase enzymes, compared with control. Chitosan significantly reduced weight loss and hydrogen peroxide and malondialdehyde levels of fruits during storage time.	[163]
Grapes immersed in chitosan (10 g/L, 0.1 g/L empty capsules,), respectively iturin A encapsulated in chitosan (IA/CS 0.1 g/L) for 10 min.	“Kyoho”	IA/CS significantly reduced decay and respiration intensity by 52.3% and 23.8%, respectively, compared to control, inhibited abscission rate, weight loss, firmness reduction, total soluble solids consumption, titratable acidity consumption, polyphenol oxidase, and peroxidase activities. Treatment suppressed decay, slowed post-harvest metabolic activity, and maintained grape quality.	[164]
Grapes immersed in 25 mg/L sodium selenite and 1.0% chitosan mixture for 2 min.	“Red Globe”	Selenium–chitosan treatment slowed down browning, prevented reduction of total phenolic, flavonoids, and anthocyanin in the grapes	[165]

## Data Availability

Not applicable.
